# Supplementary material for: Could teacher-perceived parental interest be an important factor in understanding how education relates to later physiological health? A life course approach
Source: PLoS One. 2021 Jun 17;16(6):e0252518. doi: 10.1371/journal.pone.0252518 (PMC8211281; doi:10.1371/journal.pone.0252518)
Supplement: S1 Table — (DOCX) [file pone.0252518.s001.docx]

# **S1 Table: Sensitivity analyses: parental interest in 4 categories on complete-case data for (A) men and (B) women**

|  | **Model 1** |  | **Model 2** |  | **Model 3** |  | **Model 4** |  | **Model 5** |  | **Model 6** |  |
| --- | --- | --- | --- | --- | --- | --- | --- | --- | --- | --- | --- | --- |
| 1. Men | **Coeff. [ CI 95%]** | ***P-value*** | **Coeff. [ CI 95%]** | ***P-value*** | **Coeff. [ CI 95%]** | ***P-value*** | **Coeff. [ CI 95%]** | ***P-value*** | **Coeff. [ CI 95%]** | ***P-value*** | **Coeff. [ CI 95%]** | ***P-value*** |
| **Parental interest 7 years (n=1 395)** |  |  |  |  |  |  |  |  |  |  |  |  |
| Very interested | ref |  | ref |  | ref |  | ref |  | ref |  | ref |  |
| Both some/little interest | **0.32 [0.11; 0.53]** | ***0.003*** | 0.11 [-0.12; 0.33] | *0.358* | -0.01 [-0.25; 0.22] | *0.924* | -0.01 [-0.25; 0.22] | 0.923 | -0.04 [-0.28; 0.19] | *0.737* | -0.04 [-0.27; 0.18] | *0.704* |
| Both or one parent over concerned | 0.001 [-0.54; 0.54] | *0.997* | 0.08 [-0.46; 0.61] | *0.784* | 0.07 [-0.47; 0.61] | *0.796* | 0.08 [-0.46; 0.62] | 0.767 | 0.06 [-0.47; 0.6] | *0.816* | -0.03 [-0.55; 0.49] | *0.911* |
| Discordant | 0.24 [-0.19; 0.68] | *0.273* | 0.18 [-0.25; 0.61] | *0.422* | 0.1 [-0.33; 0.53] | *0.651* | 0.1 [-0.33; 0.53] | 0.653 | 0.08 [-0.35; 0.51] | *0.718* | 0.10 [-0.31; 0.52] | *0.632* |
| **Parental interest 11 years (n=1 408)** |  |  |  |  |  |  |  |  |  |  |  |  |
| Very interested | ref |  | ref |  | ref |  | ref |  | ref |  | ref |  |
| Both some/little interest | **0.35 [0.14; 0.57]** | ***0.002*** | 0.19 [-0.05; 0.42] | *0.125* | 0.10 [-0.14; 0.35] | *0.405* | 0.10 [-0.14; 0.35] | *0.405* | 0.08 [-0.17; 0.33] | *0.516* | 0.11 [-0.13; 0.35] | *0.376* |
| Both or one parent over concerned | -0.02 [-0.44; 0.4] | *0.928* | -0.07 [-0.48; 0.35] | *0.747* | -0.10 [-0.52; 0.31] | *0.632* | -0.11 [-0.52; 0.31] | *0.618* | -0.08 [-0.50; 0.34] | *0.705* | -0.10 [-0.51; 0.31] | *0.637* |
| Discordant | 0.14 [-0.21; 0.49] | *0.441* | 0.03 [-0.32; 0.39] | *0.854* | -0.004 [-0.36; 0.35] | *0.983* | 0.01 [-0.34; 0.37] | *0.948* | 0.02 [-0.33; 0.38] | *0.896* | 0.02 [-0.33; 0.36] | *0.926* |
| **Parental interest 16 years (n=1 219)** |  |  |  |  |  |  |  |  |  |  |  |  |
| Very interested | ref |  | ref |  | ref |  | ref |  | ref |  | ref |  |
| Both some/little interest | **0.41 [0.18; 0.63]** | ***<0.001*** | **0.25 [0.0005; 0.49]** | ***0.05*** | 0.17 [-0.09; 0.42] | *0.198* | 0.17 [-0.09; 0.42] | *0.204* | 0.10 [-0.15; 0.36] | *0.424* | 0.04 [-0.22; 0.29] | *0.785* |
| Both or one parent over concerned | 0.15 [-0.37; 0.67] | *0.567* | 0.18 [-0.34; 0.70] | *0.493* | 0.12 [-0.4; 0.64] | *0.657* | 0.13 [-0.39; 0.65] | *0.636* | 0.15 [-0.37; 0.67] | *0.574* | 0.10 [-0.42; 0.61] | *0.713* |
| Discordant | 0.17 [-0.31; 0.65] | *0.480* | 0.13 [-0.34; 0.61] | *0.580* | 0.05 [-0.43; 0.53] | *0.833* | 0.06 [-0.42; 0.54] | *0.814* | 0.03 [-0.45; 0.51] | *0.909* | -0.06 [-0.54; 0.41] | *0.794* |
| **Parental interest (7-16y) (n=1 971)** |  |  |  |  |  |  |  |  |  |  |  |  |
| Both interested | ref |  | ref |  | ref |  | ref |  | ref |  | ref |  |
| Low/No interest | **0.33 [0.15; 0.5]** | ***<0.001*** | 0.12 [-0.07; 0.31] | *0.206* | 0.01 [-0.19; 0.21] | *0.899* | 0.01 [-0.19; 0.21] | *0.917* | -0.04 [-0.24; 0.16] | *0.669* | -0.05 [-0.24; 0.15] | *0.642* |

|  | **Model 1** | | **Model 2** | | **Model 3** | | **Model 4** | | **Model 5** | | **Model 6** | |
| --- | --- | --- | --- | --- | --- | --- | --- | --- | --- | --- | --- | --- |
| 1. Women | **Coeff. [ CI 95%]** | ***P-value*** | **Coeff. [ CI 95%]** | ***P-value*** | **Coeff. [ CI 95%]** | ***P-value*** | **Coeff. [ CI 95%]** | ***P-value*** | **Coeff. [ CI 95%]** | ***P-value*** | **Coeff. [ CI 95%]** | ***P-value*** |
| **Parental interest 7 years (n=1 381)** |  |  |  |  |  |  |  |  |  |  |  |  |
| Very interested | ref |  | ref |  | ref |  | ref |  | ref |  | ref |  |
| Both some/little interest | **0.59 [0.36; 0.82]** | ***<0.001*** | **0.30 [0.05; 0.55]** | ***0.019*** | 0.19 [-0.07; 0.45] | *0.155* | 0.18 [-0.08; 0.44] | 0.166 | 0.14 [-0.12; 0.4] | *0.281* | 0.13 [-0.12; 0.39] | *0.314* |
| Both or one parent over concerned | -0.10 [-0.76; 0.57] | *0.776* | -0.22 [-0.88; 0.43] | *0.507* | -0.2 [-0.86; 0.45] | *0.540* | -0.21 [-0.87; 0.44] | 0.526 | -0.14 [-0.79; 0.51] | *0.676* | -0.21 [-0.86; 0.43] | *0.520* |
| Discordant | **0.54 [0.09; 0.99]** | ***0.019*** | 0.43 [-0.02; 0.88] | *0.06* | 0.38 [-0.06; 0.83] | *0.093* | 0.38 [-0.07; 0.82] | 0.101 | 0.35 [-0.1; 0.79] | *0.126* | 0.37 [-0.07; 0.81] | *0.103* |
| **Parental interest 11 years (n=1 393)** |  |  |  |  |  |  |  |  |  |  |  |  |
| Very interested | ref |  | ref |  | ref |  | ref |  | ref |  | ref |  |
| Both some/little interest | **0.70 [0.46; 0.94]** | ***<0.001*** | **0.34 [0.08; 0.6]** | ***0.01*** | 0.24 [-0.04; 0.51] | *0.089* | 0.22 [-0.05; 0.49] | *0.114* | 0.18 [-0.10; 0.45] | *0.207* | 0.15 [-0.12; 0.42] | *0.269* |
| Both or one parent over concerned | 0.25 [-0.28; 0.78] | *0.359* | 0.2 [-0.32; 0.73] | *0.45* | 0.17 [-0.35; 0.7] | *0.518* | 0.17 [-0.36; 0.69] | *0.536* | 0.14 [-0.38; 0.66] | *0.589* | 0.11 [-0.4; 0.63] | *0.668* |
| Discordant | **0.52 [0.15; 0.89]** | ***0.006*** | 0.33 [-0.04; 0.70] | *0.081* | 0.27 [-0.11; 0.64] | *0.166* | 0.28 [-0.10; 0.65] | *0.146* | 0.24 [-0.13; 0.62] | *0.206* | 0.24 [-0.13; 0.61] | *0.201* |
| **Parental interest 16 years (n=1 247)** |  |  |  |  |  |  |  |  |  |  |  |  |
| Very interested | ref |  | ref |  | ref |  | ref |  | ref |  | ref |  |
| Both some/little interest | **0.57 [0.33; 0.81]** | ***<0.001*** | 0.25 [-0.01; 0.51] | *0.063* | 0.15 [-0.13; 0.43] | *0.289* | 0.14 [-0.14; 0.41] | *0.338* | 0.07 [-0.21; 0.35] | *0.618* | -0.0005 [-0.27; 0.27] | *0.997* |
| Both or one parent over concerned | 0.08 [-0.66; 0.81] | *0.835* | 0.09 [-0.64; 0.82] | *0.809* | 0.01 [-0.72; 0.74] | *0.981* | 0.02 [-0.71; 0.75] | *0.951* | 0.02 [-0.71; 0.75] | *0.952* | -0.01 [-0.73; 0.71] | *0.976* |
| Discordant | 0.30 [-0.25; 0.85] | *0.289* | 0.22 [-0.32; 0.76] | *0.429* | 0.19 [-0.36; 0.73] | *0.498* | 0.18 [-0.36; 0.73] | *0.512* | 0.18 [-0.36; 0.73] | *0.504* | 0.17 [-0.37; 0.7] | *0.542* |
| **Parental interest (7-16y) (n=2 040)** |  |  |  |  |  |  |  |  |  |  |  |  |
| Both interested | ref |  | ref |  | ref |  | ref |  | ref |  | ref |  |
| Low/No interest | **0.69 [0.5; 0.87]** | ***<0.001*** | **0.41 [0.21; 0.61]** | ***<0.001*** | **0.32 [0.11; 0.53]** | ***0.003*** | **0.31 [0.09; 0.52]** | ***0.005*** | **0.25 [0.04; 0.46]** | ***0.020*** | **0.23 [0.02; 0.44]** | ***0.034*** |
