# Supplementary material for: Could teacher-perceived parental interest be an important factor in understanding how education relates to later physiological health? A life course approach
Source: PLoS One. 2021 Jun 17;16(6):e0252518. doi: 10.1371/journal.pone.0252518 (PMC8211281; doi:10.1371/journal.pone.0252518)
Supplement: S2 Table — (DOCX) [file pone.0252518.s002.docx]

**S2 Table: Sensitivity analyses imputing parental interest measurement vs complete-case parental interest measurement for men and women**

|  |  |  |  |
| --- | --- | --- | --- |
|  | **Coeff. [ IC 95%]** | | **P-value** |
| *Men: imputed (n=4 075)* |  |  |  |
| Parental interest |  |  |  |
| Both interested | ref | |  |
| Low/No interest | -0.01 [-0.16; 0.14] | | 0.866 |
| *Men: complete-case (n=1 971)* |  |  |  |
| Parental interest |  |  |  |
| Both interested | ref | |  |
| Low/No interest | -0.05 [-0.24; 0.15] | | 0.642 |
| *Women: imputed (n=4 056)* |  |  |  |
| Parental interest |  |  |  |
| Both interested | ref | |  |
| Low/No interest | 0.18 [0.01; 0.35] | | 0.041 |
| *Women: complete-case (n=2 040)* |  |  |  |
| Parental interest |  |  |  |
| Both interested | ref | |  |
| Low/No interest | 0.23 [0.02; 0.44] | | 0.034 |
