# Supplementary material for: Could teacher-perceived parental interest be an important factor in understanding how education relates to later physiological health? A life course approach
Source: PLoS One. 2021 Jun 17;16(6):e0252518. doi: 10.1371/journal.pone.0252518 (PMC8211281; doi:10.1371/journal.pone.0252518)
Supplement: S3 Table — Abbreviations and symbols: n = number of people; med = median; p25 = 25e percentile; p75 = 75e percentile. Values corresponding to the categories of allostatic load: Low: [0–2]; Medium: [3–4]; High: [5–12]. *P-values were calculated using a chi-squared test for categorical variables and Student t-test for the continuous variable. (DOCX) [file pone.0252518.s003.docx]

### **S3 Table: Descriptive characteristic of the non-imputed subsample (n= 8 113) among men and women.**

| **Variable** | | **Level** | **Men**  **n=4057 (50.01%)** | **Women**  **n=4076 (49.99%)** | **Total n=8113(100%)** | **P-value*** |
| --- | --- | --- | --- | --- | --- | --- |
| **Allostatic load (44y)** | | Low | 1793 (44.20%) | 1824 (44.97%) | 3617 (44.58%) | <0.001 |
|  | | Medium | 1386 (34.16%) | 1235 (30.45%) | 2621 (32.31%) |  |
|  | | High | 878 (21.64%) | 997 (24.58%) | 1875 (23.11%) |  |
|  | |  |  |  |  |  |
| **Parental interest (7-16y)** | | Both interested | 1912 (47.13%) | 1906 (46.99%) | 3818 (47.06%) | 0.313 |
|  | | Low/No interest | 1703 (41.98%) | 1666 (41.07%) | 3369 (41.53%) |  |
|  | | Missing | 442 (10.89%) | 484 (11.93%) | 926 (11.41%) |  |
| *Childhood socioeconomic confounders* |  | |  |  |  |  |
| **Parental SEC (birth)** | | I & II | 748 (18.44%) | 706 (17.41%) | 1454 (17.92%) | 0.749 |
|  | | IIINM | 380 (9.37%) | 391 (9.64%) | 771 (9.50%) |  |
|  | | IIIM | 1913 (47.15%) | 1911 (47.12%) | 3824 (47.13%) |  |
|  | | IV&V | 768 (18.93%) | 797 (19.65%) | 1565 (19.29%) |  |
|  | | Missing | 248 (6.11%) | 251 (6.19%) | 499 (6.15%) |  |
|  | |  |  |  |  |  |
| **Material living conditions (7y)** | | Advantaged | 2495 (61.50%) | 2463 (60.72%) | 4958 (61.11%) | 0.146 |
|  | | Disadvantaged | 938 (23.12%) | 1007 (24.83%) | 1945 (23.97%) |  |
|  | | Missing | 624 (15.38%) | 586 (14.45%) | 1210 (14.91%) |  |
|  | |  |  |  |  |  |
| **Father's level of education (7y)** | | Left school ≥15y | 873 (21.52%) | 906 (22.34%) | 1779 (21.93%) | 0.653 |
|  | | Left school <14y | 2574 (63.45%) | 2539 (62.60%) | 5113 (63.02%) |  |
|  | | Missing | 610 (15.04%) | 611 (15.06%) | 1221 (15.05%) |  |
|  | |  |  |  |  |  |
| **Mother's level of education (birth)** | | Left school ≥15y | 1023 (25.22%) | 1025 (25.27%) | 2048 (25.24%) | 0.997 |
|  | | Left school <14y | 2800 (69.02%) | 2796 (68.93%) | 5596 (68.98%) |  |
|  | | Missing | 234 (5.77%) | 235 (5.79%) | 469 (5.78%) |  |
|  | |  |  |  |  |  |
| **Reading activities (7y)** | | Every week | 2030 (50.04%) | 1966 (48.47%) | 3996 (49.25%) | 0.001 |
|  | | Occasionally | 1083 (26.69%) | 1241 (30.60%) | 2324 (28.65%) |  |
|  | | Hardly ever | 409 (10.08%) | 356 (8.78%) | 765 (9.43%) |  |
|  | | Missing | 535 (13.19%) | 493 (12.15%) | 1028 (12.67%) |  |
|  | |  |  |  |  |  |
| **Outdoor activities (7y)** | | Most weeks | 3076 (75.82%) | 3172 (78.21%) | 6248 (77.01%) | 0.033 |
|  | | Occasionally/hardly ever | 452 (11.14%) | 396 (9.76%) | 848 (10.45%) |  |
|  | | Missing | 529 (13.04%) | 488 (12.03%) | 1017 (12.54%) |  |
|  | |  |  |  |  |  |
| **Place in the sibling (7 y)** | | Single child | 294 (7.25%) | 305 (7.52%) | 599 (7.38%) | 0.561 |
|  | | Elder | 1068 (26.32%) | 1071 (26.41%) | 2139 (26.37%) |  |
|  | | ≥ 2 | 2178 (53.68%) | 2204 (54.34%) | 4382 (54.01%) |  |
|  | | Missing | 517 (12.74%) | 476 (11.74%) | 993 (12.24%) |  |
|  | |  |  |  |  |  |
| **ACEs (7-16y)** | | No | 2721 (67.07%) | 2775 (68.42%) | 5496 (67.74%) | 0.302 |
|  | | Yes | 1032 (25.44%) | 1007 (24.83%) | 2039 (25.13%) |  |
|  | | Missing | 304 (7.49%) | 274 (6.76%) | 578 (7.12%) |  |
|  | |  |  |  |  |  |
| **Health problems in childhood (7-16y)** | | No | 2988 (73.65%) | 3102 (76.48%) | 6090 (75.06%) | 0.013 |
|  | | Yes | 1040 (25.63%) | 929 (22.90%) | 1969 (24.27%) |  |
|  | | Missing | 29 (0.71%) | 25 (0.62%) | 54 (0.67%) |  |
|  | |  |  |  |  |  |
| **Cognitive skills (7y)** | | Score: med [p25-p75] | 7\|6-9] | 7\|6-8] | 7 [6-9] | 0.147 |
|  | | Missing | 473 (11.66%) | 428 (10.55%) | 901 (11.11 %) |  |
| *Intermediate lifecourse variables* |  | |  |  |  |  |
| **Education level (23y)** | | A level | 855 (21.07%) | 817 (20.14%) | 1672 (20.61%) | <0.001 |
|  | | O level | 1324 (32.63%) | 1598 (39.40%) | 2922 (36.02%) |  |
|  | | No level | 1280 (31.55%) | 1140 (28.11%) | 2420 (29.83%) |  |
|  | | Missing | 598 (14.74%) | 501 (12.35%) | 1099 (13.55%) |  |
|  | |  |  |  |  |  |
| **Malaise inventory (23y)** | | No psychological distress | 3336 (82.23%) | 3196 (78.80%) | 6532 (80.51%) | <0.001 |
|  | | Psychological distress | 120 (2.96%) | 357 (8.80%) | 477 (5.88%) |  |
|  | | Missing | 601 (14.81%) | 503 (12.40%) | 1104 (13.61%) |  |
|  | |  |  |  |  |  |
| **Sense of personal control (33y)** | | Internal | 3033 (74.76%) | 3140 (77.42%) | 6173 (76.09%) | <0.001 |
|  | | External | 324 (7.99%) | 451 (11.12%) | 775 (9.55%) |  |
|  | | Missing | 700 (17.25%) | 465 (11.46%) | 1165 (14.36%) |  |
|  | |  |  |  |  |  |
| **Occupational social class (33y)** | | I & II | 1418 (34.95%) | 1173 (28.92%) | 2591 (31.94%) | <0.001 |
|  | | IIINM | 359 (8.85%) | 1252 (30.87%) | 1611 (19.86%) |  |
|  | | IIIM | 1103 (27.19%) | 255 (6.29%) | 1358 (16.74%) |  |
|  | | IV&V | 483 (11.91%) | 748 (18.44%) | 1231 (15.17%) |  |
|  | | Missing | 694 (17.11%) | 628 (15.48%) | 1322 (16.29%) |  |
|  | |  |  |  |  |  |
| **Wealth (33y)** | | Owner highest price | 660 (16.27%) | 699 (17.23%) | 1359 (16.75%) | 0.001 |
|  | | Owner high price | 685 (16.88%) | 682 (16.81%) | 1367 (16.85%) |  |
|  | | Owner median price | 666 (16.42%) | 719 (17.73%) | 1385 (17.07%) |  |
|  | | Owner lowest price | 674 (16.61%) | 663 (16.35%) | 1337 (16.48%) |  |
|  | | Not owner | 797 (19.65%) | 843 (20.78%) | 1640 (20.21%) |  |
|  | | Missing | 575 (14.17%) | 450 (11.09%) | 1025 (12.63%) |  |
|  | |  |  |  |  |  |
| **Smoking (42y)** | | Non-smoker | 1763 (43.46%) | 1813 (44.70%) | 3576 (44.08%) | <0.001 |
|  | | Ex smoker | 1038 (25.59%) | 985 (24.29%) | 2023 (24.94%) |  |
|  | | Smoker < 10 cig./day | 289 (7.12%) | 293 (7.22%) | 582 (7.17%) |  |
|  | | Smoker 10 to 19 cig./day | 305 (7.52%) | 418 (10.31%) | 723 (8.91%) |  |
|  | | Smoker more than 20 cig./day | 519 (12.79%) | 428 (10.55%) | 947 (11.67%) |  |
|  | | Missing | 143 (3.52%) | 119 (2.93%) | 262 (3.23%) |  |
|  | |  |  |  |  |  |
| **Alcohol consumption (42y)** | | Moderate | 2183 (53.81%) | 2560 (63.12%) | 4743 (58.46%) | <0.001 |
|  | | Abstinent | 662 (16.32%) | 1126 (27.76%) | 1788 (22.04%) |  |
|  | | High | 1070 (26.37%) | 251 (6.19%) | 1321 (16.28%) |  |
|  | | Missing | 142 (3.50%) | 119 (2.93%) | 261 (3.22%) |  |
|  | |  |  |  |  |  |
| **Physical activity (42y)** | | Active | 2617 (64.51%) | 2602 (64.15%) | 5219 (64.33%) | <0.001 |
|  | | Moderate | 380 (9.37%) | 298 (7.35%) | 678 (8.36%) |  |
|  | | Inactive | 917 (22.60%) | 1036 (25.54%) | 1953 (24.07%) |  |
|  | | Missing | 143 (3.52%) | 120 (2.96%) | 263 (3.24%) |  |

Abbreviations and symbols: n = number of people; med = median; p25 = 25e percentile; p75 = 75e percentile. Values corresponding to the categories of allostatic load: Low: [0-2]; Medium: [3-4]; High: [5-12]. *P-values were calculated using a chi-squared test for categorical variables and Student t-test for the continuous variable.
