# Supplementary material for: Could teacher-perceived parental interest be an important factor in understanding how education relates to later physiological health? A life course approach
Source: PLoS One. 2021 Jun 17;16(6):e0252518. doi: 10.1371/journal.pone.0252518 (PMC8211281; doi:10.1371/journal.pone.0252518)
Supplement: S1 File — (DOCX) [file pone.0252518.s004.docx]

# **S1 File: Detail on variable constructions**

## **Early life confounders’ variables**

Parental SEC

Parental SEC was constructed via a questionnaire completed at birth using the British Registrar General's social class system (RGSC) classifying the father's social class and if this was not available, the social class of the mother's father was used.

Material living conditions

Parents provided information at ages 7, 11 and 16 on whether they were living in a house/apartment or in a bedroom/caravan/other, if they were using their own indoor toilet or if it was outdoor/shared, if they had their own bathroom or if it was absent/shared, if they had their own kitchen or if it was absent/shared, if they had access to hot water or if it was shared, and how many people were sleeping in the same room (≤ 1.5 or >1.5). Using these items, we created a binary variable describing material living conditions as being advantaged or disadvantaged.

The level of education of the mother and father

The only measure of mother's education available was self-reported at birth and the measure of father's education was self-reported at age 7, providing information on whether the mother and the father had left school after legal minimal age (15y) or whether they had pursued their education.

Parenting practices

Parenting practices including reading to the child and outdoor activities were measured at age 7. Parents provided information on the regularity of outings with their child (e.g. for walks, outings, picnics, visits, shopping) and how often the mother and/or father are read to, or read with, the child (every week/occasionally/hardly ever). Two variables were created, “Reading activities” with 3 categories “Frequent, Occasionally, Hardly ever” and “Outdoor activities” with 2 categories “Frequent/Occasionally, Hardly ever” due to their distribution.

Adverse childhood experiences

Adverse childhood experiences (ACEs) was built according previous work using the National Child Development Study and in accordance with the initial deﬁnition of ACEs, as a set of psychological trauma, abuse, living conditions, out of the child’s control, that can generate chronic stress responses in the child’s immediate environment [1,2]. Information was extracted via variables collected at ages 7, 11, and 16 from survey asked to the child's parents and teacher or completed by health visitor. Type of adversity were divided into six categories: child has ever been in public, voluntary care services or foster care/child appears undernourished, dirty/the child lived in a household where a family member was in prison or on probation or is in contact with probation service; the child has ever been to prison or been on probation/the child has been separated from their father or mother due to death, divorce, or separation/household has contact with mental health services; family member has mental illness/family member has alcohol abuse problem [1,3]. According these data, we constructed a binary variable that reveals the absence or presence of an adversity.

Health problems in childhood

Health problems in childhood can have an effect on educational trajectories [4] but also on subsequent health [5]. In order to create a binary variable describing whether or not the child had health problems, we used the medical information provided by the school doctor or the child’s parents on congenital conditions, moderate/severe disabilities, chronic respiratory or circulatory conditions, sensory impairments and special schooling at 7, 11 and 16 years of age.

Birth order

Birth order was included, to take into account the child’s position in relation to their siblings. Research shows that the presence of older siblings is associated with relatively better mental health than having younger siblings [6] though according to Montgomery et al. stress resilience is adversely affected by having older brothers [7]. Furthermore, birth order can impact educational achievement with earlier-born children were found to be more likely than later-born children to obtain educational qualifications [8]. We used information provided by parents at age 7 to construct a birth order variable (eldest/2nd position or more/single child).

Child’s cognitive ability

To measure the child’s cognitive ability, which can both influence education level [9] and health behaviors [10], we used a measure derived from the Copy-a-Design test [11] available in our data collected at age 7. It measures a child's capacity to reproduce geometrical figures, scores range between 0 and 12, with higher scores relating to better fine motor skills and the child's ability to adapt to the school environment by following instructions.

## **Intermediate lifecourse variables**

Educational pathway

Educational attainment was created using information given by cohort members at age 23 when they provided information on the last educational diploma they had obtained. Education was coded into three categories: passed the Advanced Level (A level–12 years of education)/passed the Ordinary Level (O levels–10 years of education)/ No qualifications. The O levels represents the minimum leaving school age and corresponds to age 15-16y. The A level represents the final high school diploma in the UK, at around 18 years of age.

Socioeconomic position

We investigated socio-economic position at age 33 by measuring the occupational social class of the cohort members using data previously classified according to the RGSC and by measuring a wealth variable constructed from information on home ownership and the price of the home adjusted for economic inflation in the year of purchase and then divided into quartiles.

Psychological/psychosocial pathways

- Malaise

Malaise is “a generalized feeling of discomfort, illness, uneasiness, fatigue or lack of well-being, often associated with a disease state” [12]. We used the malaise inventory to measure psychological distress at the age of 23. The malaise inventory has been found to have acceptable internal validity in different socio-economic groups in the NCDS sample [13].

- Sense of personal control

An individual who is having an "external" sense of personal control will tend to attribute an external causality to the events he will experience, while an individual who is having an "internal" sense of personal control will tend to attribute a specific causality to his person, with an appropriate understanding and a capacity to manage this events [14]. To construct the binary variable measuring internal or external sense of personal control, we used self-reported data by the cohort members at age 33, based on the Rutter' scale [15].

Health behaviors pathway

Adult life-style variables are available at different sweeps in the cohort. However, for our study, we used adult variables at the age of 42 as a proxy for behavioral patterns in adulthood. The health behavior variables included here were: alcohol consumption at 42 years, based on the national recommendation of UK health department in 1995 (normal drinking (women: between 1 and 21 units in the previous week, men: between 1 and 28 units in the previous week)/abstinence (reported not consuming any alcohol in the previous week)/heavy drinking (women: more than 21 units in the previous week, men: more than 28 units in the previous week ) [16]; smoking status at 42 years (Non-smoker/Ex smoker/Smoker < 10 cig,/day/Smoker 10 to/19 cig,/day/Smoker more than 20 cig,/day), and physical activity at 42 years (Physically active/Moderately active/Inactive).

[1] Solís CB, Kelly-Irving M, Fantin R, Darnaudéry M, Torrisani J, Lang T, et al. Adverse childhood experiences and physiological wear-and-tear in midlife: Findings from the 1958 British birth cohort. Proc Natl Acad Sci 2015;112:E738–46. https://doi.org/10.1073/pnas.1417325112.

[2] Kelly-Irving M, Lepage B, Dedieu D, Bartley M, Blane D, Grosclaude P, et al. Adverse childhood experiences and premature all-cause mortality. Eur J Epidemiol 2013;28:721–34. https://doi.org/10.1007/s10654-013-9832-9.

[3] Felitti VJ, Anda RF, Nordenberg D, Williamson DF, Spitz AM, Edwards V, et al. Relationship of Childhood Abuse and Household Dysfunction to Many of the Leading Causes of Death in Adults. Am J Prev Med 1998;14:245–58. https://doi.org/10.1016/S0749-3797(98)00017-8.

[4] Isohanni I, Jones PB, Järvelin M-R, Nieminen P, Rantakallio P, Jokelainen J, et al. Educational consequences of mental disorders treated in hospital. A 31-year follow-up of the Northern Finland 1966 Birth Cohort. Psychol Med 2001;31:339–49. https://doi.org/10.1017/S003329170100304X.

[5] Jefferis BJMH, Power C, Hertzman C. Birth weight, childhood socioeconomic environment, and cognitive development in the 1958 British birth cohort study. BMJ 2002;325:305. https://doi.org/10.1136/bmj.325.7359.305.

[6] Lawson DW, Mace R. Siblings and childhood mental health: evidence for a later-born advantage. Soc Sci Med 1982 2010;70:2061–9. https://doi.org/10.1016/j.socscimed.2010.03.009.

[7] Montgomery S, Bergh C, Udumyan R, Eriksson M, Fall K, Hiyoshi A. Sex of older siblings and stress resilience. Longitud Life Course Stud 2018;9:447–55. https://doi.org/10.14301/llcs.v9i4.486.

[8] Bu F. Examining sibling configuration effects on young people’s educational aspiration and attainment. Adv Life Course Res 2016;27:69–79. https://doi.org/10.1016/j.alcr.2015.09.003.

[9] Gottfredson LS, Deary IJ. Intelligence Predicts Health and Longevity, but Why? Curr Dir Psychol Sci 2004;13:1–4. https://doi.org/10.1111/j.0963-7214.2004.01301001.x.

[10] Batty GD, Deary IJ, Macintyre S. Childhood IQ in relation to risk factors for premature mortality in middle‐aged persons: the Aberdeen Children of the 1950s study. J Epidemiol Community Health 2007;61:241–7. https://doi.org/10.1136/jech.2006.048215.

[11] Lacey RE, Cable N, Stafford M, Bartley M, Pikhart H. Childhood socio-economic position and adult smoking: are childhood psychosocial factors important? Evidence from a British birth cohort. Eur J Public Health 2011;21:725–31. https://doi.org/10.1093/eurpub/ckq179.

[12] Scully C. 4 - Signs and symptoms. In: Scully C, editor. Scullys Med. Probl. Dent. Seventh Ed., Oxford: Churchill Livingstone; 2014, p. 97–122. https://doi.org/10.1016/B978-0-7020-5401-3.00004-7.

[13] Rodgers B, Pickles A, Power C, Collishaw S, Maughan B. Validity of the Malaise Inventory in general population samples. Soc Psychiatry Psychiatr Epidemiol 1999;34:333–41. https://doi.org/10.1007/s001270050153.

[14] Becker GS. Investment in Human Capital: A Theoretical Analysis. J Polit Econ 1962;70:9–49. https://doi.org/10.1086/258724.

[15] Wang L, Lv M. Internal-External Locus of Control Scale. In: Zeigler-Hill V, Shackelford TK, editors. Encycl. Personal. Individ. Differ., Cham: Springer International Publishing; 2020, p. 2339–43. https://doi.org/10.1007/978-3-319-24612-3_41.

[16] Department of Health. Sensible Drinking The Report of an Inter-Departmental Working Group. London, UK: Department of Health: 1995.
