# Supplementary material for: Could teacher-perceived parental interest be an important factor in understanding how education relates to later physiological health? A life course approach
Source: PLoS One. 2021 Jun 17;16(6):e0252518. doi: 10.1371/journal.pone.0252518 (PMC8211281; doi:10.1371/journal.pone.0252518)
Supplement: S2 File — (DOCX) [file pone.0252518.s005.docx]

**S2 File: Direct and indirect effect**

To disentangle and quantify the direct and indirect effect for parents' interest in their child's education as perceived by teachers (PI) on allostatic load (AL), we proceeded to different steps of analysis.

- **Step 1: Total effect analysis.**

We estimated the total effect of *PI* on *AL* using a linear regression, adjusted for baseline confounders (denoted *L*):

$\mathbb{E}\left( AL | L, PI \right)=\mu_{1}+ \beta_{L.1}L+ \beta_{PI.1}PI$. $E\left( \mathrm{AL} | L,PI \right)=\mu_{1}+\beta_{L.1}L+\beta_{AL.1}\mathrm{AL}$The total effect was estimated by ${TE}_{PI}= \hat{\beta}_{PI.1}$ (See figure 4A).

- **Step 2: Estimation of the direct effect of PI on AL**.

Assuming no unmeasured confounders between mediators and *AL*, nor between *PI* and *AL*, we used the model 6 described earlier to estimate the direct effect of parental interest on *AL*, not mediated by any of the mediators under study i.e. educational level, denoted *EDU;* $\mathrm{EDU}$psychosocial/psychological variables, denoted *PSY*$\mathrm{PSY}$ (sense of personal control, malaise); social position, denoted *SEP* (occupational social class and wealth); health behaviors variables, denoted *HB*$\mathrm{HB}$ (smoking, alcohol consumption, physical activity):

$\mathbb{E}\left( AL | L,PI,EDU,PSY,SEP,HB \right)=\mu_{2}+ \beta_{L.2}L+ \beta_{PI.2}PI + \beta_{EDU.2}EDU+ \beta_{PSY.2}PSY+\beta_{SEP.2}SEP+ \beta_{HB.2}HB$

The direct effect of *PI* on *AL* was estimated by ${DE}_{PI}= \hat{\beta}_{PI.2}$ (See figure 4B).

- **Step 3: Analysis of the natural direct effect of PI on AL not going through education level**

To analyze the natural direct effect of *PI* on *AL*, not going through education level (*EDU*), we used the model 3 described earlier: $\mathbb{E}\left( AL | L,PI,EDU \right)=\mu_{3}+ \beta_{L.3}L+ \beta_{PI.3}PI + \beta_{EDU.3}EDU$.

Assuming no unmeasured confounders between *EDU* and *AL*, nor between *PI* and *AL*, and no interaction, the natural direct effect of *PI* on *AL* can be estimated by ${NDE}_{PI|EDU}= \hat{\beta}_{PI.3}$. This natural direct effect corresponds to the combination of two pathways between *PI* and *AL*, not going through *EDU* in Figure 1C: {*PI→AL*} and {*PI→other mediators→AL*}.

- **Step 4: Analysis of the natural indirect effect going through education level.**

We estimated the natural indirect effect of *PI* on *AL*, through educational level, by the difference: ${NIE}_{PI \to EDU}={TE}_{PI}-{NDE}_{PI} = \hat{\beta}_{PI.1}- \hat{\beta}_{PI.3}$

This natural indirect effect corresponds to the combination of two indirect pathways going through educational level in figure 1B: {*PI→EDU→AL*} and {*PI→EDU→other mediators→AL*} (See figure 4C).

- **Step 5: Analysis of the indirect effect of PI on AL going through other mediators but not through education level.**

We deduced the indirect effect of PI on AL going through other mediators but not through EDU by the difference ${IE}_{PI \to OtherM}={NDE}_{PI|EDU}- {DE}_{PI}= \hat{\beta}_{PI.3}- \hat{\beta}_{PI.1}$ (See figure 4D).

- **Step 6: Quantification of a percentage of effect**

Finally each of these direct and indirect estimated effects was reported in percentage of the total effect ${TE}_{PI}$.

**Fig 4: Representation of different direct and indirect estimated effects of PI on AL**
