# Supplementary material for: Could teacher-perceived parental interest be an important factor in understanding how education relates to later physiological health? A life course approach
Source: PLoS One. 2021 Jun 17;16(6):e0252518. doi: 10.1371/journal.pone.0252518 (PMC8211281; doi:10.1371/journal.pone.0252518)
Supplement: Attachment — Submitted filename: PONE-D-20-18411_reviewer (1).pdf [file pone.0252518.s006.pdf]

PLOS ONE

Could teacher-perceived parental interest be an important factor in understanding how education relates to later physiological health? A life course approach

--Manuscript Draft--

**Manuscript Number:**	PONE-D-20-18411
**Article Type:**	Research Article
**Full Title:**	Could teacher-perceived parental interest be an important factor in understanding how education relates to later physiological health? A life course approach
**Short Title:**	Teacher-perceived parental interest: factor in understanding how education relates to physiological health ?
**Corresponding Author:**	Camille Joannès<br>Université Paul Sabatier<br>Toulouse, Midi-Pyrénées FRANCE
**Keywords:**	Education gradient; Parental interest; Allostatic load; Embodiment; Lifecourse
**Abstract:**	Education is associated with later health, and notably with allostatic load (AL), an indicator of physiological health measuring the cost of adapting to stressful conditions. However, education is itself constructed by a number of upstream variables. We examined the origins of educational attainment through the prism of interactions between families and school i.e. teacher-perceived parental interest in their child education (PI). This study aims to examine whether PI is associated with AL, and whether education is a possible mediator of the relationship.<br>We used data from the National Child Development Study. Linear regression analyses on a total of 7850 participants revealed that people whose parents were considered to be uninterested in their education by their teacher had a higher AL on average in midlife (men: β=0.4 [0.28; 0.54]; women: β = 0.7 [0.52; 0.88]). PI and AL were related along three pathways: education, material/financial, and behavioural. Among women, 45% of the effect remained unexplained by mediation pathways.<br>This work may provide evidence that dissonance between family and educational cultures in childhood is associated with subsequent physiological health in mid-life.
**Order of Authors:**	Camille Joannès
	Raphaële Castagné
	Benoit Lepage
	Cyrille Delpierre
	Michelle Kelly-Irving
**Additional Information:**	
**Question**	**Response**
**Financial Disclosure**<br><br>Enter a financial disclosure statement that describes the sources of funding for the work included in this submission. Review the submission guidelines for detailed requirements. View published research articles from *PLOS ONE* for specific examples.<br><br>This statement is required for submission and **will appear in the published article** if the submission is accepted. Please make sure it is accurate.	The author(s) received no specific funding for this work.

Powered by Editorial Manager® and ProduXion Manager® from Aries Systems Corporation

**Unfunded studies**<br>Enter: *The author(s) received no specific funding for this work.*<br><br>**Funded studies**<br>Enter a statement with the following details:<br>• Initials of the authors who received each award<br>• Grant numbers awarded to each author<br>• The full name of each funder<br>• URL of each funder website<br>• Did the sponsors or funders play any role in the study design, data collection and analysis, decision to publish, or preparation of the manuscript?<br>• **NO** - Include this sentence at the end of your statement: *The funders had no role in study design, data collection and analysis, decision to publish, or preparation of the manuscript.*<br>• **YES** - Specify the role(s) played.<br><br>* typeset	
**Competing Interests**<br><br>Use the instructions below to enter a competing interest statement for this submission. On behalf of all authors, disclose any competing interests that could be perceived to bias this work—acknowledging all financial support and any other relevant financial or non-financial competing interests.<br><br>This statement **will appear in the published article** if the submission is accepted. Please make sure it is accurate. View published research articles from *PLOS ONE* for specific examples.	The authors have declared that no competing interests exist.

Powered by Editorial Manager® and ProduXion Manager® from Aries Systems Corporation

**NO authors have competing interests**<br><br>Enter: *The authors have declared that no competing interests exist.*<br><br>**Authors with competing interests**<br><br>Enter competing interest details beginning with this statement:<br><br>*I have read the journal's policy and the authors of this manuscript have the following competing interests: [insert competing interests here]*<br><br>* typeset	
**Ethics Statement**<br><br>Enter an ethics statement for this submission. This statement is required if the study involved:<br><br>• Human participants<br>• Human specimens or tissue<br>• Vertebrate animals or cephalopods<br>• Vertebrate embryos or tissues<br>• Field research<br><br>Write "N/A" if the submission does not require an ethics statement.<br><br>General guidance is provided below. Consult the submission guidelines for detailed instructions. **Make sure that all information entered here is included in the Methods section of the manuscript.**	Written informed consent was obtained from parents for childhood measurements and ethical approval for the adult data collection was obtained from the National Research Ethics Advisory Panel. NCDS data are open access datasets available to non-profit research organizations. Ethical approval for the age 45 year survey was given by the South East Multicentre Research Ethics Committee.

Powered by Editorial Manager® and ProduXion Manager® from Aries Systems Corporation

**Format for specific study types**<br><br>**Human Subject Research (involving human participants and/or tissue)**<br>• Give the name of the institutional review board or ethics committee that approved the study<br>• Include the approval number and/or a statement indicating approval of this research<br>• Indicate the form of consent obtained (written/oral) or the reason that consent was not obtained (e.g. the data were analyzed anonymously)<br><br>**Animal Research (involving vertebrate animals, embryos or tissues)**<br>• Provide the name of the Institutional Animal Care and Use Committee (IACUC) or other relevant ethics board that reviewed the study protocol, and indicate whether they approved this research or granted a formal waiver of ethical approval<br>• Include an approval number if one was obtained<br>• If the study involved *non-human primates*, add *additional details* about animal welfare and steps taken to ameliorate suffering<br>• If anesthesia, euthanasia, or any kind of animal sacrifice is part of the study, include briefly which substances and/or methods were applied<br><br>**Field Research**<br><br>Include the following details if this study involves the collection of plant, animal, or other materials from a natural setting:<br>• Field permit number<br>• Name of the institution or relevant body that granted permission	
**Data Availability**<br><br>Authors are required to make all data underlying the findings described fully available, without restriction, and from the time of publication. PLOS allows rare exceptions to address legal and ethical concerns. See the PLOS Data Policy and FAQ for detailed information.	No - some restrictions will apply

Powered by Editorial Manager® and ProduXion Manager® from Aries Systems Corporation

A Data Availability Statement describing where the data can be found is required at submission. Your answers to this question constitute the Data Availability Statement and **will be published in the article**, if accepted.<br><br>**Important:** Stating 'data available on request from the author' is not sufficient. If your data are only available upon request, select 'No' for the first question and explain your exceptional situation in the text box.<br><br>Do the authors confirm that all data underlying the findings described in their manuscript are fully available without restriction?	
**Describe where the data may be found in full sentences. If you are copying our sample text, replace any instances of XXX with the appropriate details.**<br><br>• If the data are **held or will be held in a public repository**, include URLs, accession numbers or DOIs. If this information will only be available after acceptance, indicate this by ticking the box below. For example: *All XXX files are available from the XXX database (accession number(s) XXX, XXX.).*<br>• If the data are all contained **within the manuscript and/or Supporting Information files**, enter the following: *All relevant data are within the manuscript and its Supporting Information files.*<br>• If neither of these applies but you are able to provide **details of access elsewhere**, with or without limitations, please do so. For example:<br><br>*Data cannot be shared publicly because of [XXX]. Data are available from the XXX Institutional Data Access / Ethics Committee (contact via XXX) for researchers who meet the criteria for access to confidential data.*<br><br>*The data underlying the results presented in the study are available from (include the name of the third party*	NCDS data are available by registering on the UK data service repository https://ukdataservice.ac.uk/. However, we are not allowed to share them.<br>All files are available from the 7 database (Persistent identifier (DOI): 10.5255/UKDA-SN-5560-4 and 10.5255/UKDA-SN-5594-2)<br>We used biomedical data, which is sensitive data, accessible via the same site by requesting a special license.

and contact information or URL).<br>• This text is appropriate if the data are owned by a third party and authors do not have permission to share the data.<br><br>* typeset	
Additional data availability information:	

Powered by Editorial Manager® and ProduXion Manager® from Aries Systems Corporation

Manuscript Click here to access/download;Manuscript;Manuscript.docx

Could teacher-perceived parental interest be an important factor in understanding how education relates to later physiological health? A life course approach

Camille Joannès[1*], Raphaële Castagné[1], Benoit Lepage[1], Cyrille Delpierre[1], Michelle Kelly-Irving[1,2]

[1] Equity research team, LEASP UMR1027, Inserm, Université Toulouse III Paul Sabatier, Toulouse, France

[2] Interdisciplinary Federal Research Institute on Health & Society (IFERISS), Université Toulouse III Paul Sabatier, Toulouse, France

*Corresponding author

E-mail: camille.joannes@univ-tlse3.fr

Keywords

Education gradient; Parental interest; Allostatic load; Embodiment; Lifecourse

Abstract

Education is associated with later health, and notably with allostatic load (AL), an indicator of physiological health measuring the cost of adapting to stressful conditions. However, education is itself constructed by a number of upstream variables. We examined the origins of educational attainment through the prism of interactions between families and school i.e. teacher-perceived parental interest in their child's education (PI). This study aims to examine whether PI is associated with AL, and whether education is a possible mediator of the relationship.

We used data from the National Child Development Study. Linear regression analyses on a total of 7850 participants revealed that people whose parents were considered to be uninterested in their education by their teacher had a higher AL on average in midlife (men: β=0.4 [0.28; 0.54]; women: β = 0.7 [0.52; 0.88]). PI and AL were related along three pathways: education, material/financial, and behavioural. Among women, 45% of the effect remained unexplained by mediation pathways.

This work may provide evidence that dissonance between family and educational cultures in childhood is associated with subsequent physiological health in mid-life.

Introduction

One of the most consistent findings in the field of social epidemiology is that educational attainment is associated with health. Across countries, and over time, lower educational attainment has been associated with poorer health outcomes (1). These associations are often explained by the fact that well educated people are less likely to experience the harsh material conditions or psychosocial distress caused by economic hardship and tend to have healthier lifestyles compared to the less educated (2). However, the mechanisms through which education relates to health, remain poorly understood.

The concept of embodiment rests upon a key set of mechanisms likely to underlie the relationship between social variables like education and health. This concept “refers to how we, like any living organism, literally incorporate, biologically, the world in which we live, including our societal and ecological circumstances"(3). Allostatic load (AL) is an example of how endogenous mechanisms, by which daily interactions and relationships are perceived and interpreted by the central nervous system, may over time lead to multi-system physiological wear-and-tear. It measures the consequence of a prolonged activation of the stress response system by external challenges, leading to physiological imbalances across systems (4). Previous research has shown that AL is associated with physical and functioning decline, cardiovascular events, and mortality (5,6)

Life course research indicates that the dynamic processes of adaptive allostasis most likely begin in early life (7,8). Early life socioeconomic conditions are associated with physiological wear-and-tear through educational attainment (9). As such, educational attainment “is an excellent marker of the ‘healthfulness’ of accumulated childhood experience’’(10) as the social environment in early life, partly operating through education, may be associated with different physiological responses, leading to physiological wear-and-tear in more disadvantaged social

groups, which in turn affects health. The question is, which elements of the early life environment upstream of education are likely to be involved in the embodiment dynamic, leading to physiological wear-and-tear?

This early life social environment, is complex and can be examined through the three dimensions of economic capital, social capital and cultural capital (11,12). Economic capital refers to the material resources and financial support, social capital concerns interpersonal support whereas cultural capital exists in three forms: incorporated (e.g. values, skills), objectivized (e.g. cultural goods, books) and institutionalized (e.g. educational level). Initially, through the family social sphere, these three dimensions are important facets of early childhood socialization. However, the family social sphere, meets the educational social sphere when a child attends school and if families have been socialized outside of the normative educational structure, they potentially do not or cannot adapt to the school environment (13). This dissonance that may be experienced by children exposed to home and school social environments that are socio-culturally distant from each other, may be an early stressor for the child, and lead to a solicitation of their physiological stress response system. In turn, this physiological response may affect subsequent physiological health.

Parental interest in their child's education could be a variable of interest to examine how the relationship between the home and school environments in early life affects physiological wear-and-tear, through educational attainment. Indeed, parental interest has been identified as a determinant of education success (14,15). Furthermore, it has positive effects on psychosocial adjustment (16) and later mental health (17). Teacher's evaluations provide one perspective of the situation which may partly reflect the position and viewpoint of the educational institution in terms of their compliance with academic requirements and potentially capture the tension between home and school environments experienced by some children.

We hypothesize that teacher-perceived parental interest (PI) may capture early life stressors linked to dissonance between the home and school environment and thus be related to physiological wear-and-tear, partly through education. In this study, we take a life course approach to (i) test whether PI is associated with AL, and (ii) we explored four pathways through which PI may be differentially embodied during childhood, adolescence and early adulthood, leading to physiological wear-and-tear, as measured by AL.

Materials and methods

2.1. Study population

Data are from the 1958 National Child Development Study (NCDS), which included all live births in Great Britain during one week in 1958 (n = 18,555). The NCDS has been described in detail elsewhere (18). Subsequent data collections (sweeps) were carried out on cohort members aged 7y and 55y. Between 44 and 45 years of age, a biomedical survey was conducted including a self-reported questionnaire, blood and saliva samples as well as anthropometric measurements. The sample inclusion and exclusion criteria for this analysis is described in Fig. 1. Written informed consent was obtained from parents for childhood measurements and ethical approval for the adult data collection was obtained from the National Research Ethics Advisory Panel. NCDS data are open access datasets available to non-profit research organizations. Ethical approval for the age 45 year survey was given by the South East Multicentre Research Ethics Committee.

Figure 1. Diagram of inclusion and exclusion criteria for the analysis from the biomedical survey of the NCDS 58.

2.2. Allostatic load at 44y

The AL score was constructed based on previous work using the NCDS using the initial definition of AL (19) : in order to represent four physiological systems, 14 available biomarkers were used: the neuroendocrine system (salivary cortisol t1, salivary cortisol t1–t2); the immune and inflammatory system (insulin-like growth factor-1 (IGF1), C-reactive protein (CRP), fibrinogen, Immunoglobulin E (IgE)); the metabolic system (high-density lipoprotein (HDL),

low-density lipoprotein (LDL), triglycerides, glycosylated hemoglobin (HbA1C)); the cardiovascular and respiratory systems: (systolic blood pressure (SBP), diastolic blood pressure (DBP), heart rate, peak expiratory flow). Using sex-specific quartiles, each biomarker was dichotomized into "high" (coded as 1) and "low" (coded as 0) risk. The sum of these 14 dichotomized biomarkers resulted in an overall AL score ranging from 0 to 14, where a higher score represented a higher AL. We also recoded AL into a 3 category variable where a score of 0-2 was considered to be “low”, 3-4 as “middle ”, and 5-14 as “high” as used previously within this cohort (20).

2.3. Teacher-perceived parental interest

PI was measured at age 7, 11 and 16 using information provided by the child’s teachers. The teacher was asked to report the level of interest of each parent in their child’s education, with four possible answers: Overly concerned; Very interested; Some interest; and Little interest. Based on this, we created a new binary variable for PI aiming to identify parents “interested” or with ”low/no interest” in their child’s education We grouped the “overly concerned” and “very interested” categories together to represent the “interested” category, while grouping the “some interest” with “little interest” categories together to represent “low/no interest ”. We hypothesized that interest from both parents at any one age belongs to the category “interested”. However, if only one of the parents was considered to be interested or if neither were, we considered this to belong to the category “low/no interest”. We conducted a sensitivity analyze for studying the stability of PI, using a series of regression analyses to identify whether changes to the ungrouped categories (Overly concerned; Very interested; Some interest; and Little interest) had an effect on AL. We observed no change to the results (S1 Table A).

2.4. Childhood socioeconomic confounders

We measured early life economic capital using parental socioeconomic classification of occupations (SEC), via a questionnaire completed at birth (I-professional occupations & II-intermediate occupations/III-skilled occupations (non-manual)/III-partly skilled occupations (manual)/IV-partly skilled occupations & V-unskilled occupations) and using information on material living conditions, collected at age 7, 11 and 16 (advantaged/disadvantaged).
Cultural capital was measured using parental educational attainment self-reported at birth (both parents left school ≥ 15year/both parents left school <14 year/mother lefts school ≥15 year and father <14 year/father lefts school ≥15 year and mother <14 year) and parenting practices including reading to the child and outdoor activities, measured at age 7 ("Frequent/Occasionally, Hardly ever) (21).
Other prior confounding variables potentially associated with PI and AL were selected. At ages 7, 11, and 16, a binary adverse childhood experiences variable (ACEs) was constructed, as well as a binary childhood pathologies variable. Using data collected at 7y, a birth order variable was created (Single child/Elder/2nd place or more), and an assessment of the child's cognitive ability (Copy-a-Design test where scores range between 0 and 12). See S2 File for more information about early life confounders' variables.

2.5. Intermediate lifecourse variables

In order to determine whether any observed associations between PI and AL were due to subsequent adult intermediate factors, the following mediating factors were added to the models: respondent's educational attainment at 23 y (A level/O level/no qualification), socioeconomic status [respondent's occupational social class at 33 y

(Favored/Median/Disadvantaged); respondent's financial status at 33 y using a wealth variable based on information about home ownership and the price of the house adjusted for economic inflation of the year of purchase (not owner/Q1—owner lowest price/owner-Q2/owner Q3/owner-Q4)]; psychological/psychosocial status [malaise inventory at 23y (No psychological distress/ psychological distress); sense of personal control (SOC) at 33y (Internal/external)]; health behaviors at 42 y were considered as a proxy for behavioral patterns in adulthood included self-reported physical activity, alcohol consumption and smoking status. See S2 File for more information about intermediate lifecourse variables.

2.6. Statistical analysis

Our analyses were stratified by sex. Behavior at school differs between the two genders: girls being more compliant with institutional rules, facilitating the teacher's task, boys are more frequently in conflict between academic expectations and their socially recognized particularities. Also, this allows us to take into account sex/gender differences in health.

First, descriptive and bivariate statistics were carried out using the Chi2 test, considering AL as a categorical variable in three groups, in order to ascertain any association between the covariates and AL. Second, to study the association between *PI* and AL, regression coefficients and 95% confidence intervals (CI) were estimated using linear regressions where AL was entered as a continuous variable. We compared regression coefficients across nested models to observe the change in effect according to subsequent adjustments.

To study the link between teachers' perceptions of parental interest and AL

- Model 1: Linear regression between PI and AL

- Model 2: Model 1 plus baseline confounders characterizing parental cultural capital (level of parental education, lecture and outdoor activities) and economic capital (SEC and material living conditions) plus other early-life confounders (ACEs, place among the sibling, health problems in childhood, initial cognition).

To qualify pathways mediating the relationship between teachers' perceptions of parental interest and AL.

Applying the general approach of Baron & Kenny (22) to study the role intermediate variables in models adjusted for mediators, we defined the sets of mediators according to the temporal and causal assumptions of the life course approach.

We assumed the set health behaviors (at 42y) came after and could be influenced by socioeconomic status (at 33y), which came after and could be influenced by psychosocial condition (at 23y), which came after and could be influenced by education (at 23y), which came after and could be influenced by teachers' perceptions of parental interest. In these models, we considered that the last set of mediators had an important mediating role if the change of the regression coefficient characterizing the association between teachers' perceptions of parental interest (*PI*) and AL was large and if these mediators were associated to AL.

- Model 3: Model 2 plus educational level, denoted *EDU* (level of education)
- Model 4: Model 3 plus psychosocial/psychological variables, denoted *PSY* (SOC, malaise)
- Model 5: Model 4 plus socioeconomic status/financial variables, denoted *SEP* (social class, incomes)
- Model 6: Model 5 plus health behaviors variables, denoted *HB* (smoking, alcohol consumption, sportive practice)

Finally, to disentangle and quantify the direct and indirect effect for PI on AL, we carried-out different steps in both men and women (S3 File), based on previous studies analyzing mediation (23).

In order to control for potential biases due to missing data, multivariate analyses were conducted on the imputed database using the ICE method of the imputation program available on Stata ®v14. Twenty imputations were performed assuming that the data were missing at random (MAR). Comparisons were then made between full case multivariate analyses and multivariate analyses based on imputation estimates, indicating selection biases in the full case sample (S4 Table B).

Results

Descriptive and bivariate analyses from the bivariate analyses by AL group of the nonimputed subsample are given in Table 1 in me[illegible]d women. The majority of our population (78% in men and 75% in women) had a low [0-2] or medium [3-4] AL. Additionally, 47% of the cohort members' two parents were perceived by the teacher as taking interest in their child's education, while 42% were described as uninterested or not very interested.

In general men and women with a high AL at 44y were more likely to have parents described as having a low interest in their education by the teacher and to have no level of education at 23y. Other childhood variables associated with AL in both men and women were: parent's education, parent's social class, material living conditions, adverse childhood experiences and cognitive skills. For men, outdoor activities, birth order, and health problems were also associated with adulthood AL. Regarding intermediate variables in adulthood, graded associations were also observed with AL such as SEP, income, SOC, health behavior for both sexes and malaise only for women.

Bivariate analyses by PI are reported in Table 2. PI was associated with all childhood variables, and all intermediate variables. In relation to our hypothesis on the educational pathway, female and male participants who were hardly ever read to as children, hardly ever had outdoors activities and whose mothers had left school before the age of 14, who lived in unfavorable conditions and whose parents had a low SEC, were more likely to have parents who were described as uninterested or not very interested in their education by the teacher.

Table 1: Bivariate statistics on the subsample (n= 8,113) of the distribution of AL according to confounding and intermediate variables in men and in women.

	Allostatic load at 44y								
	**Men n (%) n=4,075 (50%)**				**Women n (%) n=4056 (50%)**				**Total n (%)**
	**Low**	**Medium**	**High**	***p***	**Low**	**Medium**	**High**	***p***	
	1,793 (44%)	1,386 (34%)	878 (22%)		1,824 (44,97%)	1,235 (30%)	997 (25%)		
**Teachers' perceptions of parental interest (7-16y)**									
Both interested	930 (52%)	634 (46%)	348 (40%)	***<0,001***	989 (54%)	555 (45%)	362 (36%)	***<0,001***	3,818 (47%)
Low/No interest	698 (39%)	578 (42%)	427 (49%)		654 (36%)	524 (42%)	488 (49%)		3,369 (42%)
Missing	165 (9%)	174 (13%)	103 (12%)		181 (10%)	156 (13%)	147 (15%)		926 (11%)
Childhood socioeconomic confounders									
**Parental education level (birth)**									
Both parents left school ≥15y	216 (12%)	139 (10%)	93 (11%)	***<0,001***	210 (12%)	135 (11%)	80 (8%)	***<0,001***	873 (11%)
Both parents left school <14y	351 (20%)	272 (20%)	159 (18%)		367 (20%)	244 (20%)	195 (20%)		1,588 (20%)
Mother left school ≥ 15y, father <14 y	298 (17%)	186 (13%)	89 (10%)		342 (19%)	163 (13%)	93 (9%)		1,171 (14%)
Father left school ≥ 15y, mother <14y	885 (49%)	736 (53%)	505 (58%)		857 (47%)	660 (53%)	597 (60%)		4,240 (52%)
Missing	43 (2%)	53 (4%)	32 (4%)		48 (3%)	33 (3%)	32 (3%)		241 (3%)
**Reading activities (7y)**									
Every week	920 (51%)	687 (50%)	423 (48%)	*0,328*	893 (49%)	590 (48%)	483 (48%)	*0,773*	3,996 (49%)
Occasionally	492 (27%)	365 (26%)	249 (28%)		556 (30%)	396 (32%)	309 (31%)		2,367 (29%)
Hardly ever	169 (9%)	132 (10%)	85 (10%)		160 (9%)	91 (7%)	85 (9%)		722 (9%)
Missing	212 (12%)	202 (15%)	121 (14%)		215 (12%)	158 (13%)	120 (12%)		1,028 (13%)
**Outdoor activities (7y)**									
Most weeks	1,402 (78%)	1,017 (73%)	657 (75%)	***0,032***	1,449 (79%)	951 (77%)	772 (77%)	*0,41*	6,248 (77%)
Occasionally/hardly ever	182 (10%)	169 (12%)	101 (12%)		161 (9%)	130 (11%)	105 (11%)		848 (10%)
Missing	209 (12%)	200 (14%)	120 (14%)		214 (12%)	154 (12%)	120 (12%)		1,017 (13%)
**Parental SEC (birth)**									
I & II	405 (23%)	240 (17%)	103 (12%)	***<0,001***	414 (23%)	184 (15%)	108 (11%)	***<0,001***	1,454 (18%)

IIINM	196 (11%)	117 (8%)	67 (8%)		201 (11%)	110 (9%)	80 (8%)		771 (10%)
IIIM	805 (45%)	666 (48%)	442 (50%)		789 (43%)	613 (50%)	509 (51%)		3,824 (47%)
IV&V	295 (16%)	262 (19%)	211 (24%)		309 (17%)	248 (20%)	240 (24%)		1,565 (19%)
Missing	92 (5%)	101 (7%)	55 (6%)		111 (6%)	80 (6%)	60 (6%)		499 (6%)
**Material living conditions (7y)**									
Advantaged	1,191 (66%)	804 (58%)	500 (57%)	***<0,001***	1,158 (63%)	735 (60%)	570 (57%)	***<0,001***	4,958 (61%)
Disadvantaged	337 (19%)	360 (26%)	241 (27%)		399 (22%)	308 (25%)	300 (30%)		1,945 (24%)
Missing	265 (15%)	222 (16%)	137 (16%)		267 (15%)	192 (16%)	127 (13%)		1,210 (15%)
**Place in the siblings (7 y)**									
≥ 2	961 (54%)	741 (53%)	476 (54%)	***0,024***	1,015 (56%)	661 (54%)	528 (53%)	*0,344*	4,382 (54%)
Elder	511 (29%)	347 (25%)	210 (24%)		481 (26%)	317 (26%)	273 (27%)		2,139 (26%)
Single child	115 (6%)	103 (7%)	76 (9%)		119 (7%)	107 (9%)	79 (8%)		599 (7%)
Missing	206 (11%)	195 (14%)	116 (13%)		209 (11%)	150 (12%)	117 (12%)		993 (12%)
**ACEs (7-16y)**									
No	1,285 (72%)	891 (64%)	545 (62%)	***<0,001***	1,322 (72%)	827 (67%)	626 (63%)	***<0,001***	5,496 (68%)
Yes	399 (22%)	374 (27%)	259 (30%)		389 (21%)	320 (26%)	298 (30%)		2,039 (25%)
Missing	109 (6%)	121 (9%)	74 (8%)		113 (6%)	88 (7%)	73 (7%)		578 (7%)
**Health problems in childhood (7-16y)**									
No	1,360 (76%)	1,002 (72%)	626 (71%)	***0,014***	1,431 (78%)	923 (75%)	748 (75%)	*0,09*	6,090 (75%)
Yes	416 (23%)	376 (27%)	248 (28%)		383 (21%)	302 (24%)	244 (24%)		1,969 (24%)
Missing	17 (1%)	8 (1%)	4 (0%)		10 (1%)	10 (1%)	5 (1%)		54 (1%)
**Cognitive skills (7y)**									
Score : med [p25-p75]	8 [6-9]	7 [6-9]	7 [6-8]	***<0,001***	8 [6-9]	7 [6-8]	7 [6-8]	***<0,001***	7 [6- 9]
Missing	183 (10%)	180 (13%)	110 (13%)		188 (10%)	131 (11%)	109 (11%)		901 (11%)
Intermediate lifecourse variables									
**Education level (23y)**									
A level	565 (32%)	336 (24%)	142 (16%)	***<0,001***	522 (29%)	252 (20%)	158 (16%)	***<0,001***	1,975 (24%)
O level	661 (37%)	498 (36%)	298 (34%)		801 (44%)	541 (44%)	394 (40%)		3,193 (39%)
No level	510 (28%)	494 (36%)	387 (44%)		448 (25%)	409 (33%)	406 (41%)		2,654 (33%)
Missing	57 (3%)	58 (4%)	51 (6%)		53 (3%)	33 (3%)	39 (4%)		291 (4%)

**Malaise inventory (23y)**									
No psychological distress	1,497 (83%)	1,134 (82%)	705 (80%)	*0,156*	1,508 (83%)	951 (77%)	737 (74%)	***<0,001***	6,532 (81%)
Psychological distress	42 (2%)	45 (3%)	33 (4%)		109 (6%)	127 (10%)	121 (12%)		477 (6%)
Missing	254 (14%)	207 (15%)	140 (16%)		207 (11%)	157 (13%)	139 (14%)		1,104 (14%)
**SOC (33y)**									
Internal	1,387 (77%)	1,018 (73%)	628 (72%)	***0,012***	1,440 (79%)	960 (78%)	740 (74%)	***0.037***	6,173 (76%)
External	132 (7%)	114 (8%)	78 (9%)		187 (10%)	129 (10%)	135 (14%)		775 (10%)
Missing	274 (15%)	254 (18%)	172 (20%)		197 (11%)	146 (12%)	122 (12%)		1,165 (%)
**Occupational social class (33y)**									
Favored	713 (40%)	460 (33%)	245 (28%)	***<0,001***	609 (33%)	339 (27%)	225 (23%)	***<0,001***	2,591 (32%)
Median	620 (35%)	509 (37%)	333 (38%)		661 (36%)	470 (38%)	376 (38%)		2,969 (37%)
Disadvantaged	186 (10%)	163 (12%)	134 (15%)		289 (16%)	242 (20%)	217 (22%)		1,231 (15%)
Missing	274 (15%)	254 (18%)	166 (19%)		265 (15%)	184 (15%)	179 (18%)		1,322 (16%)
**Income (33y)**									
No income	273 (15%)	293 (21%)	231 (26%)	***<0,001***	274 (15%)	270 (22%)	299 (30%)	***<0,001***	1,640 (20%)
Q1 : very low	281 (16%)	224 (16%)	169 (19%)		281 (15%)	200 (16%)	182 (18%)		1,337 (16%)
Q2 : low	293 (16%)	232 (17%)	141 (16%)		329 (18%)	234 (19%)	156 (16%)		1,385 (17%)
Q3 : median	356 (20%)	220 (16%)	109 (12%)		350 (19%)	203 (16%)	129 (13%)		1,367 (17%)
Q4 : high	370 (21%)	203 (15%)	87 (10%)		395 (22%)	186 (15%)	118 (12%)		1,359 (17%)
Missing	220 (12%)	214 (15%)	141 (16%)		195 (11%)	142 (12%)	113 (11%)		1,025 (13%)
**Smoking (42y)**									
No/Ex smoker	1,404 (78%)	900 (65%)	497 (57%)	***<0,001***	1,391 (76%)	825 (67%)	582 (58%)	***<0,001***	5,599 (69%)
Smoker < 10 cig,/d	130 (7%)	108 (8%)	51 (6%)		143 (8%)	85 (7%)	65 (7%)		582 (7%)
Smoker ≥ 10 cig,/d	206 (11%)	319 (23%)	299 (34%)		240 (13%)	295 (24%)	311 (31%)		1,670 (21%)
Missing	53 (3%)	59 (4%)	31 (4%)		50 (3%)	30 (2%)	39 (4%)		262 (3%)
**Alcohol consumption (42y)**									
Moderate	1,078 (60%)	730 (53%)	375 (43%)	***<0,001***	1,249 (68%)	769 (62%)	542 (54%)	***<0,001***	4,743 (58%)
Abstinent	237 (13%)	233 (17%)	192 (22%)		416 (23%)	364 (29%)	346 (35%)		1,788 (22%)
High	425 (24%)	365 (26%)	280 (32%)		109 (6%)	72 (6%)	70 (7%)		1,321 (16%)
Missing	53 (3%)	58 (4%)	31 (4%)		50 (3%)	30 (2%)	39 (4%)		261 (3%)

**Sport (42y)**									
Active	1,253 (70%)	877 (63%)	487 (55%)	***<0,001***	1,252 (69%)	793 (64%)	557 (56%)	***<0,001***	5,219 (64%)
Moderate	168 (9%)	122 (9%)	90 (10%)		124 (7%)	103 (8%)	71 (7%)		678 (8%)
Inactive	318 (18%)	329 (24%)	270 (31%)		398 (22%)	308 (25%)	330 (33%)		1,953 (24%)
Missing	54 (3%)	58 (4%)	31 (4%)		50 (3%)	31 (3%)	39 (4%)		263 (3%)

Table 2: Bivariate statistics on the subsample (n= 8,113) of the distribution of PI according to confounding and intermediate variables in men and in women.

		Teachers' perceptions of parental interest (7-16y)								
		Men n (%) n=4,075 (50%)				Women n (%) n=4056 (50%)				
		Both interested<br>1,912 (47%)	Low/No interest<br>1,703 (42%)	Missing<br>442 (11%)	*p*	Both interested<br>1,906 (47%)	Low/No interest<br>1,666 (41%)	Missing<br>484 (12%)	*p*	Total n (%)
**Allostatic load (44y)**	Low	930 (49%)	698 (41%)	165 (37%)	***<0,001***	989 (52%)	654 (39%)	181 (37%)	***<0,001***	3,617 (45%)
	Medium	634 (33%)	578 (34%)	174 (39%)		555 (29%)	524 (31%)	156 (32%)		2,621 (32%)
	High	348 (18%)	427 (25%)	103 (23%)		362 (19%)	488 (29%)	147 (30%)		1,875 (23%)
**Parental education level (birth)**	Both left school ≥15y	270 (14%)	141 (8%)	37 (8%)	***<0,001***	255 (13%)	132 (8%)	38 (8%)	***<0,001***	873 (11%)
	Both left school <14y	383 (20%)	292 (17%)	107 (24%)		370 (19%)	311 (19%)	125 (26%)		1,588 (20%)
	Mother≥15y, father <14y	434 (23%)	102 (6%)	37 (8%)		469 (25%)	86 (5%)	43 (9%)		1,171 (14%)
	Father≥15y, mother <14y	786 (41%)	1,127 (66%)	213 (48%)		768 (40%)	1,105 (66%)	241 (50%)		4,240 (52%)
	Missing	39 (2%)	41 (2%)	48 (11%)		44 (2%)	32 (2%)	37 (8%)		241 (3%)
**Reading activities (7y)**	Every week	1,126 (59%)	747 (44%)	157 (36%)	***<0,001***	1,076 (56%)	715 (43%)	175 (36%)	***<0,001***	3,996 (49%)
	Occasionally	464 (24%)	523 (31%)	119 (27%)		515 (27%)	592 (36%)	154 (32%)		2,367 (29%)
	Hardly ever	145 (8%)	215 (13%)	26 (6%)		128 (7%)	177 (11%)	31 (6%)		722 (9%)
	Missing	177 (9%)	218 (13%)	140 (32%)		187 (10%)	182 (11%)	124 (26%)		1,028 (13%)
**Outdoor activities (7y)**	Most weeks	1,615 (84%)	1,202 (71%)	259 (59%)	***<0,001***	1,599 (84%)	1,257 (75%)	316 (65%)	***<0,001***	6,248 (77%)
	Occasionally/hardly ever	125 (7%)	283 (17%)	44 (10%)		128 (7%)	223 (13%)	45 (9%)		848 (10%)
	Missing	172 (9%)	218 (13%)	139 (31%)		179 (9%)	186 (11%)	123 (25%)		1,017 (13%)
**Parental SEC (birth)**	I & II	564 (30%)	135 (8%)	49 (11%)	***<0,001***	526 (28%)	132 (8%)	48 (10%)	***<0,001***	1,454 (18%)

	IIINM	223 (12%)	128 (8%)	29 (7%)		238 (12%)	119 (7%)	34 (7%)		771 (10%)
	IIIM	801 (42%)	905 (53%)	207 (47%)		788 (41%)	876 (53%)	247 (51%)		3,824 (47%)
	IV&V	229 (12%)	441 (26%)	98 (22%)		250 (13%)	445 (27%)	102 (21%)		1,565 (19%)
	Missing	95 (5%)	94 (6%)	59 (13%)		104 (5%)	94 (6%)	53 (11%)		499 (6%)
**Material living conditions (7y)**	Advantaged	1,375 (80%)	930 (68%)	190 (54%)	***<0,001***	1,342 (79%)	902 (69%)	219 (57%)	***<0,001***	4,958 (61%)
	Disadvantaged	292 (10%)	528 (20%)	118 (14%)		315 (11%)	548 (20%)	144 (18%)		1,945 (24%)
	Missing	245 (9%)	245 (13%)	134 (31%)		249 (10%)	216 (11%)	121 (25%)		1,21 (15%)
**Place in the siblings (7 y)**	≥ 2	975 (51%)	1,017 (60%)	186 (42%)	***<0,001***	965 (51%)	1,021 (61%)	218 (45%)	***<0,001***	4,382 (54%)
	Elder	612 (32%)	377 (22%)	79 (18%)		596 (31%)	377 (23%)	98 (20%)		2,139 (26%)
	Single child	155 (8%)	99 (6%)	40 (9%)		172 (9%)	89 (5%)	44 (9%)		599 (7%)
	Missing	170 (9%)	210 (12%)	137 (31%)		173 (9%)	179 (11%)	124 (26%)		993 (12%)
**ACEs (7-16y)**	No	1,534 (80%)	1,022 (60%)	165 (37%)	***<0,001***	1,531 (80%)	1,037 (62%)	207 (43%)	***<0,001***	5,496 (68%)
	Yes	287 (15%)	566 (33%)	179 (41%)		267 (14%)	541 (32%)	199 (41%)		2,039 (25%)
	Missing	91 (5%)	115 (7%)	98 (22%)		108 (6%)	88 (5%)	78 (16%)		578 (7%)
**Health problems in childhood (7-16y)**	No	1,415 (74%)	1,260 (74%)	313 (71%)	***<0,001***	1,495 (78%)	1,254 (75%)	353 (73%)		6,090 (75%)
	Yes	494 (26%)	435 (26%)	111 (25%)		409 (21%)	406 (24%)	114 (24%)	***<0,001***	1,969 (24%)
	Missing	3 (0%)	8 (0%)	18 (4%)		2 (0%)	6 (0%)	17 (4%)		54 (1%)
**Cognitive skills (7y)**	Score : med [p25-p75]	8 [6-9]	7 [6-8]	7 [6-8]	***<0,001***	8 [6-9]	7 [6-8]	7 [6-8]	***<0,001***	7 [6- 9]
	Missing	145 (8%)	183 (11%)	145 (33%)		151 (8%)	157 (9%)	(25%)		901 (11%)
**Education level (23y)**	A level	800 (42%)	169 (10%)	74 (17%)	***<0,001***	741 (39%)	111 (7%)	80 (17%)	***<0,001***	1,975 (24%)
	O level	750 (39%)	566 (33%)	141 (32%)		873 (46%)	679 (41%)	184 (38%)		3,193 (39%)
	No level	312 (16%)	875 (51%)	204 (46%)		256 (13%)	810 (49%)	197 (41%)		2,654 (33%)
	Missing	50 (3%)	93 (5%)	23 (5%)		36 (2%)	66 (4%)	23 (5%)		291 (4%)

**Malaise inventory (23y)**	No	1,623 (85%)	1,374 (81%)	339 (77%)	***<0,001***	1,610 (84%)	1,237 (74%)	349 (72%)	***<0,001***	6,532 (81%)
	Yes	36 (2%)	67 (4%)	17 (4%)		103 (5%)	201 (12%)	53 (11%)		477 (6%)
	Missing	253 (13%)	262 (15%)	86 (19%)		193 (10%)	228 (14%)	82 (17%)		1,104 (14%)
**SOC (33y)**	External	116 (6%)	169 (10%)	39 (9%)	***<0,001***	146 (8%)	231 (14%)	74 (15%)	***<0,001***	6,173 (76%)
	Internal	1,513 (79%)	1,218 (72%)	302 (68%)		1,581 (83%)	1,222 (73%)	337 (70%)		775 (10%)
	Missing	283 (15%)	316 (19%)	101 (23%)		179 (9%)	213 (13%)	73 (15%)		1,165 (%)
**Occupational social class (33y)**	Favored	926 (48%)	378 (22%)	114 (26%)	***<0,001***	745 (39%)	303 (18%)	125 (26%)	***<0,001***	2,591 (32%)
	Median	570 (30%)	727 (43%)	165 (37%)		691 (36%)	627 (38%)	189 (39%)		2,969 (37%)
	Disadvantaged	123 (6%)	288 (17%)	72 (16%)		220 (12%)	439 (26%)	89 (18%)		1,231 (15%)
	Missing	293 (15%)	310 (18%)	91 (21%)		250 (13%)	297 (18%)	81 (17%)		1,322 (16%)
**Income (33y)**	No income	287 (15%)	404 (24%)	106 (24%)	***<0,001***	268 (14%)	458 (27%)	117 (24%)	***<0,001***	1,640 (20%)
	Q1 : very low	250 (13%)	351 (21%)	73 (17%)		239 (13%)	335 (20%)	89 (18%)		1,337 (16%)
	Q2 : low	318 (17%)	287 (17%)	61 (14%)		366 (19%)	261 (16%)	92 (19%)		1,385 (17%)
	Q3 : median	400 (21%)	223 (13%)	62 (14%)		401 (21%)	219 (13%)	62 (13%)		1,367 (17%)
	Q4 : high	422 (22%)	178 (10%)	60 (14%)		447 (23%)	189 (11%)	63 (13%)		1,359 (17%)
	Missing	235 (12%)	260 (15%)	80 (18%)		185 (10%)	204 (12%)	61 (13%)		1,025 (13%)
**Smoking (42y)**	No/Ex smoker	1,420 (74%)	1,088 (64%)	293 (66%)	***<0,001***	1,480 (78%)	1,003 (60%)	315 (65%)	***<0,001***	5,599 (69%)
	Smoker < 10 cig,/d	154 (8%)	107 (6%)	28 (6%)		133 (7%)	134 (8%)	26 (5%)		582 (7%)
	Smoker ≥ 10 cig,/d	281 (15%)	445 (26%)	98 (22%)		244 (13%)	477 (29%)	125 (26%)		1,670 (21%)
	Missing	57 (3%)	63 (4%)	23 (5%)		49 (3%)	52 (3%)	18 (4%)		262 (3%)
**Alcohol consumption (42y)**	Moderate	1,143 (60%)	821 (48%)	219 (50%)	***<0,001***	1,312 (69%)	975 (59%)	273 (56%)	***<0,001***	4,743 (58%)
	Abstinent	232 (12%)	320 (19%)	110 (25%)		421 (22%)	538 (32%)	167 (35%)		1,788 (22%)
	High	480 (25%)	500 (29%)	90 (20%)		124 (7%)	101 (6%)	26 (5%)		1,321 (16%)
	Missing	57 (3%)	62 (4%)	23 (5%)		49 (3%)	52 (3%)	18 (4%)		261 (3%)

**Sport (42y)**	Active	1,305 (68%)	1,036 (61%)	276 (62%)	***<0,001***	1,282 (67%)	1,026 (62%)	294 (61%)	***<0,001***	5,219 (64%)
	Moderate	207 (11%)	134 (8%)	39 (9%)		155 (8%)	112 (7%)	31 (6%)		678 (8%)
	Inactive	342 (18%)	471 (28%)	104 (24%)		420 (22%)	475 (29%)	141 (29%)		1,953 (24%)
	Missing	58 (3%)	62 (4%)	23 (5%)		49 (3%)	53 (3%)	18 (4%)		263 (3%)

Abbreviations and symbols: n = number of people; med = median; p25 = 25e percentile; p75 = 75e percentile; statistically significant results at the 5% threshold are in bold. Values corresponding to the categories of AL: Low: [0-2]; Medium: [3-4]; High: [5-12]

Multivariate results, between PI and AL, examining the *a priori* set of confounding and intermediate factors are presented in Table 3 and 4, for men and women respectively. Men with parents perceived as uninterested or not very interested had higher AL scores at 44 years compared to those perceived as interested by the school teacher (Model 1 β= 0.41 [0.28; 0.54]). After adjustment for cultural capital, economic capital and other confounding factors in childhood, the link between PI and AL was weakened (Model 2, β= 0.18 [0.03; 0.32]) partly attributable to parental SEC, ACEs, health problems in childhood and cognitive skills. Controlling for educational attainment at 23y reduced the strength of the association between PI and AL (Model 3, β= 0.05 [-0.1; 0.2]). The association was only marginally affected when psychological status at 23 y were accounted for (Model 4, β= 0.04 [-0.1; 0.19]). Social position and income strongly affected the association (Model 5, β= 0.009 [-0.14; 0.16]) with income making a significant contribution. The association was attenuated after sequentially controlling all for time-ordered life course, SEP and health behaviours, (Model 6, β=-0.02 [-0.17; 0.13]).

A similar pattern was observed for women: women whose parents were perceived uninterested or not very interested by the teacher had higher AL scores at 44 years (Model 1, β = 0.67 [0.52; 0.81]). The association between PI and AL was attenuated after controlling for early life confounder (Model 2, β= 0.39 [0.22; 0.55]) partly explained by parental educational level and SEC and ACEs and cognitive skills. Further adjustment for educational attainment at 23 y reduced the strength of the association (Model 3, β= 0.28 [0.1; 0.46]). When psychological status was accounted for, the association between PI and AL was marginally affected (Model 4, β= 0.26 [0.08; 0.44]) but was explained by the malaise inventory. Further adjustment for social position and income slightly attenuated the association with income affecting it more strongly (Model 5, β= 0.21 [0.03; 0.39]) as well as health behaviors (Model 6, β= 0.17 [0.001;

0.35]). When all potential mediators were controlled for (Model 6), PI remained significantly associated with AL score (β= 0.17[0.001; 0.35].

Table 3: Life course multivariate linear regression between AL and PI using data obtained from multiple imputation for men (n = 3,914)

	Model 1		Model 2		Model 3		Model 4		Model 5		Model 6	
	Coeff, [IC 95%]	p	Coeff, [IC 95%]	p	Coeff, [IC 95%]	p	Coeff, [IC 95%]	p	Coeff, [IC 95%]	p	Coeff, [IC 95%]	p
**PI**												
Both interested	1		1		1		1		1		1	
Low/No interest	**0,41 [0,28; 0,54]**	**< 0,001**	**0,18 [0,03; 0,32]**	**0,016**	0,05 [-0,1; 0,2]	0,52	0,04 [-0,1; 0,19]	0,56	0,009 [-0,14; 0,16]	0,91	-0,02 [-0,17; 0,13]	0,79
**Parental education level**												
Both left school ≥15y			1		1		1		1		1	
Both left school <14y			0,09 [-0,13; 0,32]	0,42	0,09 [-0,14; 0,31]	0,44	0,09 [-0,14; 0,32]	0,43	0,08 [-0,14; 0,31]	0,48	0,06 [-0,16; 0,28]	0,57
Mother ≥ 15y, father <14y			-0,02 [-0,27; 0,24]	0,89	0,03 [-0,22; 0,29]	0,80	0,04 [-0,22; 0,29]	0,78	0,03 [-0,22; 0,28]	0,81	-0,01 [-0,26; 0,24]	0,94
Father ≥ 15y, mother <14y			0,14 [-0,06; 0,35]	0,17	0,09 [-0,12; 0,29]	0,40	0,09 [-0,12; 0,29]	0,39	0,07 [-0,14; 0,27]	0,51	0,07 [-0,13; 0,26]	0,52
**Reading activities**												
Every week			1		1		1		1		1	
Occasionally			-0,004 [-0,15; 0,14]	0,96	-0,02 [-0,17; 0,12]	0,77	-0,02 [-0,17; 0,12]	0,77	-0,01 [-0,15; 0,14]	0,93	-0,01 [-0,15; 0,13]	0,91
Hardly ever			0,03 [-0,2; 0,26]	0,78	0,02 [-0,21; 0,25]	0,86	0,02 [-0,21; 0,25]	0,87	0,03 [-0,2; 0,25]	0,80	-0,005 [-0,22; 0,21]	0,97
**Outdoor activities**												
Most weeks			1		1		1		1		1	
Occasionally/hardly ever			-0,15 [-0,35; 0,05]	0,14	-0,17 [-0,36; 0,03]	0,10	-0,17 [-0,37; 0,03]	0,10	-0,18 [-0,38; 0,01]	0,07	-0,18 [-0,37; 0,01]	0,07
**Parental SEC**												
I & II			1		1		1		1		1	
IIINM			0,02 [-0,23; 0,26]	0,88	-0,01 [-0,25; 0,24]	0,96	-0,004 [-0,25; 0,24]	0,98	0,01 [-0,23; 0,26]	0,91	-0,01 [-0,25; 0,23]	0,92
IIIM			**0,33 [0,14; 0,52]**	**0,001**	**0,27 [0,08; 0,46]**	**0,005**	**0,27 [0,08; 0,46]**	**0,005**	**0,26 [0,08; 0,45]**	**0,005**	**0,22 [0,03; 0,4]**	**0,02**
IV&V			**0,43 [0,21; 0,65]**	**< 0,001**	**0,34 [0,11; 0,56]**	**0.003**	**0,34 [0,12; 0,56]**	**0.003**	**0,3 [0,08; 0,52]**	**0,008**	**0,25 [0,04; 0,47]**	**0,022**
**Material living conditions**												
Advantaged			1		1		1		1		1	
Disadvantaged			0,15 [-0,01; 0,3]	0,07	0,14 [-0,02; 0,29]	0,09	0,13 [-0,02; 0,29]	0,10	0,12 [-0,04; 0,27]	0,15	0,11 [-0,04; 0,26]	0,15
**Place in the siblings**												
≥ 2			1		1		1		1		1	
Elder			-0,03 [-0,17; 0,11]	0,71	-0,01 [-0,15; 0,14]	0,92	-0,003 [-0,14; 0,14]	0,97	0,01 [-0,13; 0,15]	0,91	0,02 [-0,12; 0,16]	0,79
Single child			0,2 [-0,04; 0,44]	0,10	**0,24 [0,003; 0,47]**	**0,047**	**0,24 [0,004; 0,47]**	**0,046**	**0,24 [0,01; 0,48]**	**0,043**	**0,25 [0,02; 0,48]**	**0,032**
**ACEs**												
No			1		1		1		1		1	
Yes			**0,21 [0,06; 0,36]**	**0,007**	**0,17 [0,02; 0,32]**	**0,027**	**0,17 [0,01; 0,32]**	**0,03**	0,13 [-0,02; 0,29]	0,09	0,06 [-0,09; 0,21]	0,43
**Health problems**												
No			1		1		1		1		1	

Yes	**0,19 [0,05; 0,33]**	***0,008***	**0,17 [0,03; 0,31]**	***0,017***	**0,17 [0,03; 0,3]**	***0,02***	0,14 [-0,01; 0,27]	*0,06*	0,12 [-0,02; 0,26]	*0,08*
**Cognitive skills**										
Score : med [p25-p75]	**-0,07 [-0,11; -0,03]**	***< 0,001***	**-0,05 [-0,09; -0,01]**	***0,007***	**-0,05 [-0,09; -0,01]**	***0,008***	**-0,04 [-0,08; -0,01]**	***0,016***	**-0,04 [-0,07; -0,004]**	***0,029***
**Education level**										
A level			1		1		1		1	
O level			**0,28 [0,11; 0,45]**	***0,001***	**0,28 [0,11; 0,45]**	***0,001***	**0,24 [0,07; 0,41]**	***0,007***	**0,15 [-0,02; 0,32]**	***0,08***
No level			**0,57 [0,38; 0,76]**	***< 0,001***	**0,56 [0,37; 0,75]**	***< 0,001***	**0,47 [0,26; 0,67]**	***< 0,001***	**0,31 [0,1; 0,51]**	***0.003***
**Malaise inventory**										
No					1		1		1	
Yes					0,16 [-0,22; 0,53]	*0,41*	0,12 [-0,26; 0,49]	*0,54*	0,03 [-0,35; 0,4]	*0,89*
**SOC**										
External					1		1		1	
Internal					-0,12 [-0,36; 0,12]	*0,32*	-0,05 [-0,29; 0,19]	*0,67*	0,03 [-0,2; 0,27]	*0,78*
**Occupational social class**										
Favored							1		1	
Median							-0,04 [-0,21; 0,12]	*0,62*	-0,08 [-0,24; 0,08]	*0,35*
Disadvantaged							-0,005 [-0,23; 0,23]	*0,97*	-0,06 [-0,28; 0,16]	*0,61*
**Income**										
No income							1		1	
Q1 : very low							**-0,29 [-0,49; -0,09]**	***0.004***	**-0,20 [-0,39; -0,003]**	***0,047***
Q2 : low							**-0,34 [-0,56; -0,12]**	***0.002***	-0,20 [-0,42; 0,01]	*0,062*
Q3 : median							**-0,60 [-0,8; -0,4]**	***< 0,001***	**-0,40 [-0,6; -0,2]**	***< 0,001***
Q4 : high							**-0,64 [-0,85; -0,42]**	***< 0,001***	**-0,43 [-0,64; -0,23]**	***< 0,001***
**Smoking**										
No/Ex smoker									1	
Smoker < 10 cig,/d									0,03 [-0,2; 0,25]	*0,82*
Smoker ≥ 10 cig,/d									**0,82 [0,66; 0,97]**	***< 0,001***
**Alcohol consumption**										
Moderate									1	
Abstinent									**0,33 [0,17; 0,5]**	***< 0,001***
High									**0,27 [0,13; 0,41]**	***< 0,001***
**Sport**										
Active									1	
Moderate									0,12 [-0,08; 0,32]	*0,24*
Inactive									**0,34 [0,19; 0,48]**	***< 0,001***

Table 4: Life course multivariate linear regression between AL and PI using data obtained from multiple imputation for women (n = 3,936)

	Model 1		Model 2		Model 3		Model 4		Model 5		Model 6	
	**Coeff, [IC 95%]**	***p***	**Coeff, [IC 95%]**	***p***	**Coeff, [IC 95%]**	***p***	**Coeff, [IC 95%]**	***p***	**Coeff, [IC 95%]**	***p***	**Coeff, [IC 95%]**	***p***
**PI**												
Both interested	1		1		1		1		1		1	
Low/No interest	**0,67 [0,52; 0,81]**	***< 0,001***	**0,39 [0,22; 0,55]**	***< 0,001***	**0,28 [0,1; 0,46]**	***0,002***	**0,26 [0,08; 0,44]**	***0,004***	**0,209 [0,03; 0,39]**	***0,02***	**0,17 [0,001; 0,35]**	***0,049***
**Parental education level**												
Both left school ≥15y			1		1		1		1		1	
Both left school <14y			0,16 [-0,09; 0,41]	*0,22*	0,16 [-0,1; 0,41]	*0,23*	0,16 [-0,09; 0,41]	*0,22*	0,19 [-0,06; 0,44]	*0,14*	0,19 [-0,05; 0,44]	*0,12*
Mother ≥ 15y, father <14y			0,02 [-0,26; 0,29]	*0,90*	0,05 [-0,22; 0,33]	*0,70*	0,06 [-0,22; 0,33]	*0,69*	0,09 [-0,19; 0,36]	*0,54*	0,08 [-0,19; 0,36]	*0,54*
Father ≥ 15y, mother <14y			**0,34 [0,11; 0,57]**	***0,003***	**0,30 [0,07; 0,53]**	***0,01***	**0,31 [0,08; 0,54]**	***0.008***	**0,31 [0,08; 0,53]**	***0.008***	**0,31 [0,08; 0,53]**	***0.008***
**Reading activities**												
Every week			1		1		1		1		1	
Occasionally			-0,11 [-0,27; 0,05]	*0,18*	-0,11 [-0,27; 0,05]	*0,17*	-0,11 [-0,27; 0,05]	*0,18*	-0,11 [-0,27; 0,04]	*0,16*	-0,13 [-0,28; 0,02]	*0,10*
Hardly ever			-0,06 [-0,33; 0,21]	*0,68*	-0,07 [-0,34; 0,2]	*0,63*	-0,08 [-0,34; 0,19]	*0,58*	-0,08 [-0,34; 0,19]	*0,56*	-0,1 [-0,36; 0,17]	*0,47*
**Outdoor activities**												
Most weeks			1		1		1		1		1	
Occasionally/hardly ever			0,12 [-0,12; 0,37]	*0,32*	0,11 [-0,14; 0,35]	*0,38*	0,10 [-0,15; 0,34]	*0,44*	0,07 [-0,17; 0,31]	*0,54*	0,08 [-0,15; 0,32]	*0,48*
**Parental SEC**												
I & II			1		1		1		1		1	
IIINM			0,12 [-0,15; 0,4]	*0,37*	0,11 [-0,16; 0,39]	*0,42*	0,12 [-0,15; 0,39]	*0,40*	0,12 [-0,15; 0,39]	*0,40*	0,08 [-0,19; 0,35]	*0,58*
IIIM			**0,38 [0,17; 0,595]**	***< 0,001***	**0,35 [0,13; 0,56]**	***0,001***	**0,34 [0,13; 0,56]**	***0,002***	**0,3 [0,09; 0,52]**	***0.005***	**0,28 [0,07; 0,49]**	***0.008***
IV&V			**0,479 [0,23; 0,73]**	***< 0,001***	**0,42 [0,17; 0,68]**	***0,001***	**0,42 [0,16; 0,67]**	***0,001***	**0,33 [0,08; 0,59]**	***0,01***	**0,29 [0,03; 0,54]**	***0,03***
**Material living conditions**												
Advantaged			1		1		1		1		1	
Disadvantaged			0,08 [-0,08; 0,24]	*0,33*	0,07 [-0,09; 0,23]	*0,37*	0,06 [-0,09; 0,22]	*0,43*	0,04 [-0,12; 0,19]	*0,66*	0,01 [-0,15; 0,17]	*0,91*
**Place in the siblings**												
≥ 2			1		1		1		1		1	
Elder			0,12 [-0,04; 0,28]	*0,13*	0,14 [-0,02; 0,3]	*0,08*	0,15 [-0,01; 0,3]	*0,07*	0,15 [-0,01; 0,3]	*0,07*	0,15 [-0,004; 0,31]	*0,06*
Single child			0,2 [-0,08; 0,47]	*0,16*	0,23 [-0,05; 0,5]	*0,10*	0,23 [-0,04; 0,5]	*0,09*	0,23 [-0,04; 0,5]	*0,10*	**0,27 [0,01; 0,53]**	***0,04***
**ACEs**												
No			1		1		1		1		1	
Yes			**0,2 [0,02; 0,38]**	***0,03***	0,17 [-0,01; 0,34]	*0,06*	0,15 [-0,02; 0,33]	*0,09*	0,11 [-0,06; 0,28]	*0,22*	0,06 [-0,11; 0,23]	*0,47*
**Health problems**												

No	1		1		1		1		1	
Yes	0,14 [-0,02; 0,3]	*0,08*	0,13 [-0,03; 0,28]	*0,12*	0,12 [-0,04; 0,28]	*0,14*	0,11 [-0,04; 0,27]	*0,16*	0,09 [-0,06; 0,25]	*0,25*
**Cognitive skills**										
Score : med [p25-p75]	**-0,08 [-0,12; -0,05]**	***< 0,001***	**-0,07 [-0,1; -0,03]**	***0,001***	**-0,06 [-0,1; -0,02]**	***0,001***	**-0,05 [-0,09; -0,02]**	***0.005***	**-0,05 [-0,09; -0,01]**	***0.007***
**Education level**										
A level			1		1		1		1	
O level			0,14 [-0,05; 0,33]	*0,14*	0,14 [-0,05; 0,32]	*0,15*	0,06 [-0,13; 0,25]	*0,53*	0,01 [-0,18; 0,2]	*0,94*
No level			**0,46 [0,23; 0,68]**	***< 0,001***	**0,43 [0,2; 0,66]**	***< 0,001***	**0,23 [-0,01; 0,47]**	***0,06***	0,08 [-0,16; 0,32]	*0,51*
**Malaise inventory**										
No					1		1		1	
Yes					**0,36 [0,12; 0,6]**	***0,003***	**0,35 [0,11; 0,58]**	***0,004***	**0,26 [0,03; 0,49]**	***0,03***
**SOC**										
External					1		1		1	
Internal					-0,11 [-0,32; 0,1]	*0,31*	0,01 [-0,2; 0,22]	*0,95*	0,08 [-0,13; 0,29]	*0,47*
**Occupational social class**										
Favored							1		1	
Median							0,12 [-0,05; 0,3]	*0,16*	0,13 [-0,04; 0,3]	*0,13*
Disadvantaged							0,14 [-0,09; 0,38]	*0,22*	0,13 [-0,1; 0,36]	*0,27*
**Income**										
No income							1		1	
Q1 : very low							**-0,39 [-0,61; -0,18]**	***< 0,001***	**-0,31 [-0,52; -0,09]**	***0,006***
Q2 : low							**-0,62 [-0,83; -0,4]**	***< 0,001***	**-0,44 [-0,66; -0,23]**	***< 0,001***
Q3 : median							**-0,68 [-0,9; -0,46]**	***< 0,001***	**-0,50 [-0,71; -0,28]**	***< 0,001***
Q4 : high							**-0,76 [-0,99; -0,54]**	***< 0,001***	**-0,56 [-0,79; -0,34]**	***< 0,001***
**Smoking**										
No/Ex smoker									1	
Smoker < 10 cig,/d									-0,07 [-0,32; 0,18]	*0,6*
Smoker ≥ 10 cig,/d									**0,66 [0,49; 0,84]**	***< 0,001***
**Alcohol consumption**										
Moderate									1	
Abstinent									**0,34 [0,19; 0,49]**	***< 0,001***
High									0,04 [-0,23; 0,31]	*0,77*
**Sport**										
Active									1	
Moderate									0,15 [-0,1; 0,4]	*0,2*
Inactive									**0,31 [0,16; 0,46]**	***< 0,001***

The analyses of the direct and indirect effects of PI on AL are presented in Fig 2 and Fig 3. For men, the direct link between PI and AL was completely mediated, mainly by the educational pathway (72% of the total indirect effect) but also through other intermediate factors (28% of the total indirect effect), without operating through education.

For women, 55% of the link between PI and AL was mediated, through the educational pathway (28% of the total indirect effect) and by other intermediate factors (27% of the total indirect effect). A direct effect of 45% persisted after adjustment for confounding factors and mediators. For this calculation among men, we did not take into account the direct effect PI on AL because the estimation $\hat{\beta}_{PI.2} = -0{,}02\ [-0{,}17;\ 0{,}13]$ had a large confidence interval with a value close to 0.

Figure 2: Direct and indirect effect results between PI and AL for men

Figure 3: Direct and indirect effect results between PI and AL for women

Discussion

Teacher-perceived parental interest, measured when cohort members were school children, was associated with their physiological health in mid-life in both men and women. Cohort members whose parents were perceived as uninterested or not very interested in their child's education, as reported by the children's teachers, had a higher AL compared to individuals whose parents were considered by the teacher to be interested. PI appear to be related to the parent's own education level for women and parent's social class for both men and women. The association between teacher-perceived parental interest and cohort member's physiological wear-and-tear operates over the life course through intermediate pathways. Among men, 72% of the association operated through the educational pathway, and 28% through the other variables including income, smoking, alcohol consumption and physical activity. Among women, only 28% of the association operated through the education pathway, with 27% through the other variables in adulthood, including psychological variables. Much of the association (45%) was direct, and unexplained by the tested pathways. Our results are in line with other studies where parental interest in their offspring's studies was found to predict adult allostatic and may buffer against poor mental health (24,25). Our findings provide insight into understanding how educational attainment as a reflection of dynamic life course social processes relates to physiological health, but also underline that parental-interest in children's education has not been given much attention in relation to health over the life course.

It may be here that our findings underline an interplay between culture and biology (26,27) whereby a tension between a child's home and school cultural environments may lead to a physiological stress response partly mediated by the educational trajectory. This dissonance between the family and school environments may have lasting effects on the child's educational trajectory, and become reflected in their physiological functioning over time. Our results

suggest this pathway, especially for boys/men. Our findings also suggest that dissonance affects the physiological health for girls/ women directly, or through pathways that remain to be tested. Children who experience dissonance, as a chronically stressful challenge, may solicit their biological resources, experience multi-system physiological dysregulation as measured by AL, and this embodiment may represent the cost of adaptation for the children. Our findings may highlight the consequence of this hypothesized mechanism. It is also possible that the behavior of teachers towards children whose parents they consider to be less involved, could be different. Teachers easily perceive the families' economic and cultural capital once children enter school and may unconsciously show favoritism toward those students from the upper classes (28). It is possible that our findings reflect a bias or difference whereby some teachers behaved differently towards those children, which would contribute to increasing their stress and therefore impact their AL.

An important aim of our analysis was to grasp the role of intermediate factors through which teacher-perceived parental interest may affect physiological processes.

We observed two different scenarii for each gender. For women our results show that, after controlling for confounders and mediators, a sizable part of the initial effect remained unexplained. This may represent other possible pathways, or differential early life socialization and embodiment processes among girls. This suggests that intersecting domains of power including class, gender and others (race, disability etc) are likely to be at play (29,30). For men, the educational pathway had a significant and stronger effect on AL, consistent with observed differences in mortality by educational level across age groups more pronounced in men than in women (31).

Later in the life course, adult income captured a large portion of the association for women and explained the association between educational level and AL for men. Our findings suggest that

consonant relationships between family and school, captured partly by PI, could promote ascending social mobility and therefore act as a vehicle towards social advantage, that may "buffer" the effects of an initially disadvantaged socio-economic environment on AL (32,33). Furthermore, health behavior pathways appeared to explain a part of the association between PI and AL for women and effects of education level on AL for men. A consonant educational socialization could promote the embodiment of a health-relevant capital, i.e. the resources for acting in favor of health. Such consonance refers to all the “health related values, behavioral norm, knowledge and operational skills” (34). However psychological malaise was found to explain the association between PI for women and AL. Further analysis should be conducted in other cohorts to explore this association and ascertain its potential contextual specificity.

The main weakness of this study is that our variable measuring parental interest is one-sided, reflecting only the teacher’s point of view. It would have been interesting to compare this measure with parents' perceptions. However, such data were unavailable. Attrition, and selection bias, common features related to longitudinal studies also pose issue. We carried out multiple imputations, a recommended method to avoid the interpretation of biased results, allowed them to be redressed to some extent. Information and recall biases may also be present, related to the self-reported nature of the data. Furthermore, we were not able to take into account a teacher-level effect on a classroom of children. This would have required having detailed data on others children in the class or school which we did not have. With regard to alcohol consumption, we must consider that people with pathologies, but also those prone to alcohol addiction, are probably part of this group, thus biasing the results. Several years passed between the data collection sweeps and several life events probably took place between them. However, some variables in our study were measured at one given point in time, because we had only one measure available (i.e. AL, SOC, malaise), or we considered that they had a constant inertia

over time (i.e. behavior, social position). It is a regret that there is no earlier measurement of AL in order to analyze its dynamic changes over time. However, other studies show that the inertia of the measurement in adulthood remains generally constant over time, which leads us to consider that this measurement is reliable in our analysis (35). The choice of the statistical models and the variables tested are based on *a priori* theoretical and conceptual considerations. Therefore, it is possible that we overlooked variables or assumptions, other factor may contribute in the relationship between PI and AL. Lastly, NCDS 58 is a UK cohort, with unique cultural and historical aspects. It is therefore necessary to take precautions when extrapolating our results.

Despite these limitations, this study has a number of strengths. It is a longitudinal population-based study containing prospectively collected data with great detail and breadth across the life span, allowing us to control for a number variables of potential confounding and mediating factors. A parental interest measure operationalized here as a prospective variable, where information collected during childhood reported by the teacher was used to create the variable. Another important strength is in the sample size included in the biomedical survey, and the large number of biomarkers available.

Education is often used as a measure of social position, where higher educational attainment is associated with better health outcomes. Our findings suggest the importance of considering education as a product of early life interactions between family and school social sphere. In this context of coexisting social spheres, socio-cultural dissonance may occur between family and the school environment. Indeed, ‘‘The standards of the school are not neutral; their requests for parental involvement may be laden with the cultural experiences of intellectual and economic elites’’(36). Among the socially disadvantaged, who potentially don’t possess a common language and know how to negotiate the institution of school, educational

success at school may indicate a conversion of their cultural capital, described as “acculturation”. Conversely, for more socially advantaged students, this progression would be the result of the mobilization of their cultural capital heritage (37). Consequently, the dissonance between the family social sphere and the school environment may lead to an “educational acculturation”, requiring the family to assimilate to the new educational culture.

This work calls for additional studies to examine and better understand the effects of social distance between the family social sphere and the school environment in a variety of conte[illegible].

Conclusion

Teacher-perceived parental interest measured during childhood was associated with physiological wear-and-tear in mid-life in both men and women. This may be due to a physiological stress response induced from early life due to a possible dissonance between family and school cultural environments which have lasting effects on health, through pathways, including educational attainment, particularly in men. These results suggest that awareness of children’s socio-cultural environments and gender should be taken into account when developing school or educational policies. As such, understanding family educational culture, cultural capital and socioeconomic position may contribute to developing adapted public policies supporting early childhood environments to reduce social inequalities in health.

Acknowledgments

We are grateful to the Centre for Longitudinal Studies (CLS), Institute of Education for the use of the NCDS data for making them available.

References

1. Mackenbach JP, Bopp M, Deboosere P, Kovacs K, Leinsalu M, Martikainen P, et al. Determinants of the magnitude of socioeconomic inequalities in mortality: A study of 17 European countries. Health Place. 2017 Sep 1;47:44–53.

2. Ross CE, Wu C. The Links Between Education and Health. Am Sociol Rev. 1995;60(5):719–45.

3. Krieger N. Embodiment: a conceptual glossary for epidemiology. J Epidemiol Community Health. 2005 May 1;59(5):350–5.

4. McEwen BS, Stellar E. Stress and the Individual: Mechanisms Leading to Disease. Arch Intern Med. 1993 Sep 27;153(18):2093–101.

5. Juster R-P, McEwen BS, Lupien SJ. Allostatic load biomarkers of chronic stress and impact on health and cognition. Neurosci Biobehav Rev. 2010 Sep 1;35(1):2–16.

6. Karlamangla AS, Singer BH, McEwen BS, Rowe JW, Seeman TE. Allostatic load as a predictor of functional decline: MacArthur studies of successful aging. J Clin Epidemiol. 2002 Jul 1;55(7):696–710.

7. Delpierre C, Barboza-Solis C, Torrisani J, Darnaudery M, Bartley M, Blane D, et al. Origins of health inequalities: the case for Allostatic Load. Longitud Life Course Stud. 2016 Jan 19;7(1):79–103.

8. Robertson T, Popham F, Benzeval M. Socioeconomic position across the lifecourse & allostatic load: data from the West of Scotland Twenty-07 cohort study. BMC Public Health. 2014 Feb 20;14(1):184.

9. Barboza Solís C, Fantin R, Castagné R, Lang T, Delpierre C, Kelly-Irving M. Mediating pathways between parental socio-economic position and allostatic load in mid-life: Findings from the 1958 British birth cohort. Soc Sci Med. 2016 Sep;165:19–27.

10. Power C, Hertzman C. Health, well-being and coping skills. In: Developmental health and the wealth of nations. In D. P. Keating, C. Hertzman (Eds.). New York: The Guildford Press; 1999. p. 47.

11. Bourdieu P. Les trois états du capital culturel. Actes Rech En Sci Soc. 1979;30(1):3–6.

12. Bourdieu P. La Distinction, Critique sociale du jugement [Internet]. Editions de minuit. 1979 [cited 2019 Nov 20]. (Le sens commun). Available from: http://www.leseditionsdeminuit.fr/livre-La_Distinction-1954-1-1-0-1.html

13. Lahire B. Tableaux de famille. Points. 2012.

14. Flouri E, Buchanan A. Early father's and mother's involvement and child's later educational outcomes. Br J Educ Psychol. 2004 Jun;74(2):141–53.

15. Sui-Chu EH, Willms JD. Effects of Parental Involvement on Eighth-Grade Achievement. Sociol Educ. 1996;69(2):126–41.

16. Sacker A, Schoon I, Bartley M. Social inequality in educational achievement and psychosocial adjustment throughout childhood: magnitude and mechanisms. Soc Sci Med. 2002 Sep;55(5):863–80.

17. Hobcraft J. Intergenerational and Life-Course Transmission of Social Exclusion: Influences and Childhood Poverty, Family Disruption and Contact with the Police [Internet]. Rochester, NY: Social Science Research Network; 1998 Nov [cited 2020 Apr 3]. Report No.: ID 1158906. Available from: https://papers.ssrn.com/abstract=1158906

18. Power C, Elliott J. Cohort profile: 1958 British birth cohort (National Child Development Study). Int J Epidemiol. 2006 Feb 1;35(1):34–41.

19. Barboza Solís C, Fantin R, Kelly-Irving M, Delpierre C. Physiological wear-and-tear and later subjective health in mid-life: Findings from the 1958 British birth cohort. Psychoneuroendocrinology. 2016;74:24–33.

20. Castagné R, Karimi M, Chadeau-Hyam M, Vineis P, Delpierre C, Kelly-Irving M. Allostatic load and subsequent all-cause mortality: which biological markers drive the relationship? Findings from a UK birth cohort. Eur J Epidemiol. 2018 May;33(5):441–58.

21. Serre D. Le capital culturel dans tous ses états [Internet]. 2012 [cited 2019 Oct 21]. Available from: https://www.cairn.info/revue-actes-de-la-recherche-en-sciences-sociales-2012-1-page-4.htm

22. Baron RM, Kenny DA. The moderator–mediator variable distinction in social psychological research: Conceptual, strategic, and statistical considerations. J Pers Soc Psychol. 1986;51(6):1173–82.

23. Vanderweele TJ, Vansteelandt S. Conceptual issues concerning mediation, interventions and composition. Stat Interface. 2009;2(4):457–68.

24. Westerlund H, Gustafsson PE, Theorell T, Janlert U, Hammarström A. Parental academic involvement in adolescence, academic achievement over the life course and allostatic load in middle age: a prospective population-based cohort study. J Epidemiol Community Health. 2013 Jun 1;67(6):508–13.

25. Westerlund H, Rajaleid K, Virtanen P, Gustafsson PE, Nummi T, Hammarström A. Parental academic involvement in adolescence as predictor of mental health trajectories over the life course: a prospective population-based cohort study. BMC Public Health. 2015 Jul 14;15(1):653.

26. Causadias J, Telzer E, Gonzales N. The Handbook of Culture and Biology. John Wiley & Sons. The Handbook of Culture and Biology Interplay. 2018.

27. Doane LD, Sladek MR, Adam EK. An Introduction to Cultural Neurobiology: Evidence from Physiological Stress Systems. In: The Handbook of Culture and Biology [Internet]. John Wiley & Sons, Ltd; 2017 [cited 2020 Apr 14]. p. 25–254. Available from: https://onlinelibrary.wiley.com/doi/abs/10.1002/9781119181361.ch10

28. Dumais SA. Early childhood cultural capital, parental habitus, and teachers' perceptions. Poetics. 2006 Apr;34(2):83–107.

29. Crenshaw K. Demarginalizing the Intersection of Race and Sex: A Black Feminist Critique of Antidiscrimination Doctrine, Feminist Theory and Antiracist Politics. Univ Chic Leg Forum. 1989;1989:139.

30. Krieger N. Measures of Racism, Sexism, Heterosexism, and Gender Binarism for Health Equity Research: From Structural Injustice to Embodied Harm—An Ecosocial Analysis. Annu Rev Public Health. 2020;41(1):37–62.

31. Gallo V, Mackenbach JP, Ezzati M, Menvielle G, Kunst AE, Rohrmann S, et al. Social Inequalities and Mortality in Europe – Results from a Large Multi-National Cohort. PLOS ONE. 2012 Jul 25;7(7):e39013.

32. Hango D. Parental investment in childhood and educational qualifications: Can greater parental involvement mediate the effects of socioeconomic disadvantage? Soc Sci Res. 2007 Dec 1;36(4):1371–90.

33. Robertson T, Benzeval M, Whitley E, Popham F. The role of material, psychosocial and behavioral factors in mediating the association between socioeconomic position and allostatic load (measured by cardiovascular, metabolic and inflammatory markers). Brain Behav Immun. 2015 Mar;45:41–9.

34. Abel T. Cultural capital and social inequality in health. J Epidemiol Community Health. 2008 Jul 1;62(7):e13–e13.

35. Seeman TE, McEwen BS, Rowe JW, Singer BH. Allostatic load as a marker of cumulative biological risk: MacArthur studies of successful aging. Proc Natl Acad Sci. 2001 Apr 10;98(8):4770–5.

36. Lareau A. Home Advantage: Social Class and Parental Intervention in Elementary Education. Rowman & Littlefield Publishers. 1989.

37. Bourdieu P, Passeron J-C. Les héritiers : les étudiants et la culture. Editions de minuit. 1964. (Le sens commun).

Supporting information captions

- **S1 Table A**: Sensitivity analyses PI in 4 categories on complete-case data for men and women
- **S2 File:** Detail on variable constructions
- **S3 File:** Direct and indirect effect
- **S4 Table B:** Sensitivity analyses imputing PI measurement vs non-imputed PI measurement for men and women

Figure

Click here to access/download;Figure;Fig1.TIF

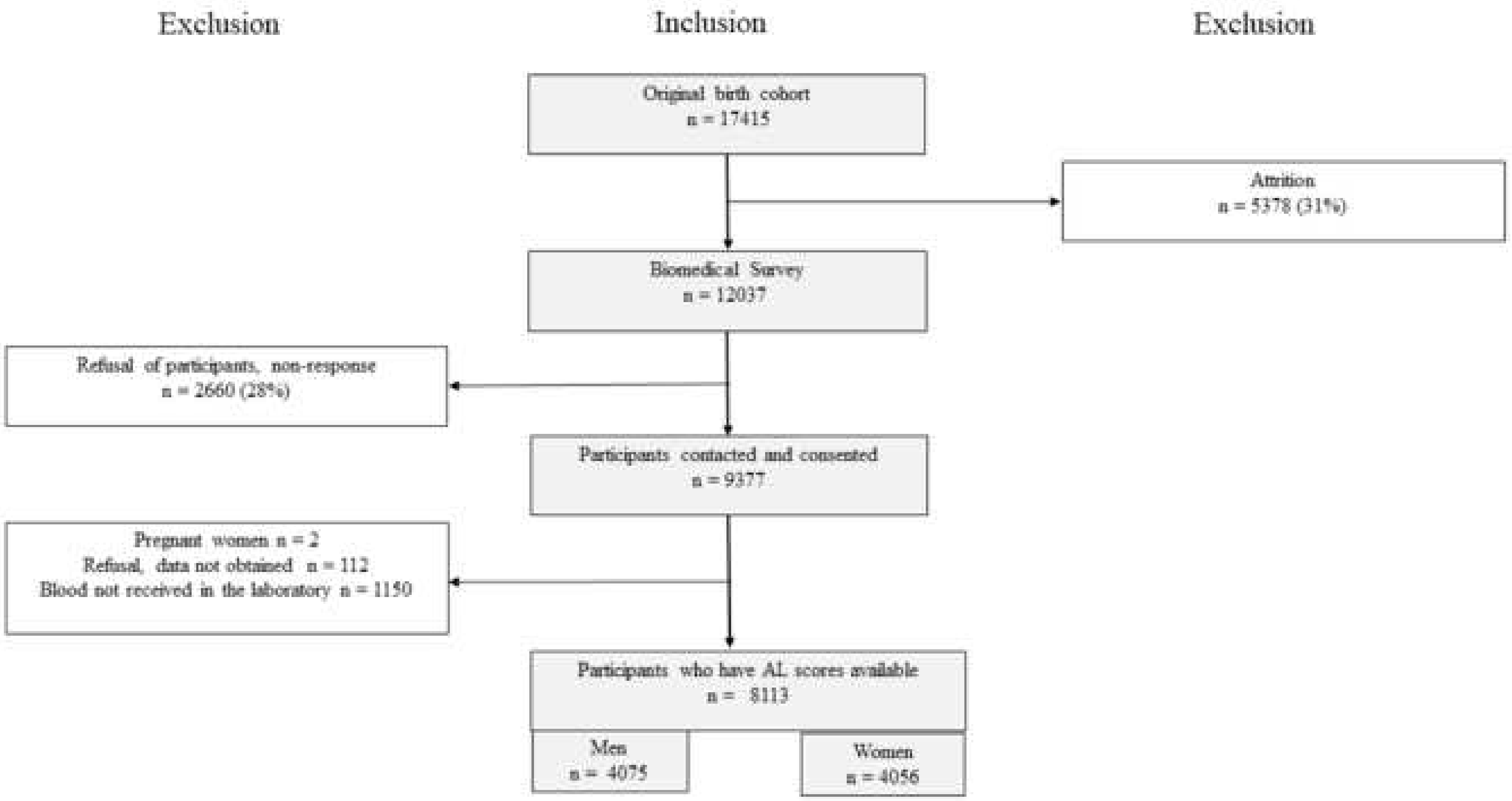

Figure 2: Direct and indirect effect results between PI and AL using data obtained from multiple imputation for men (n = 3,914)

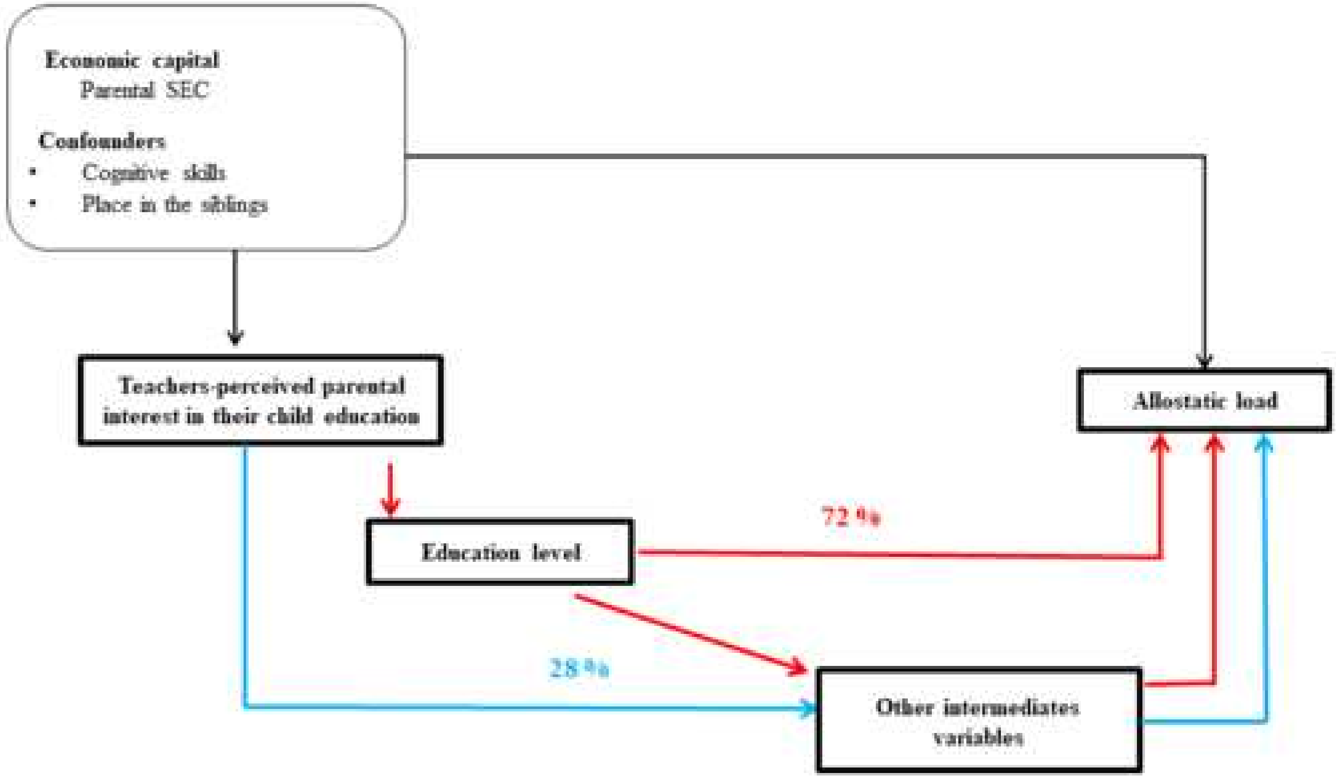

Figure 3: Direct and indirect effect results between PI and AL using data obtained from multiple imputation for women (n=3,936)

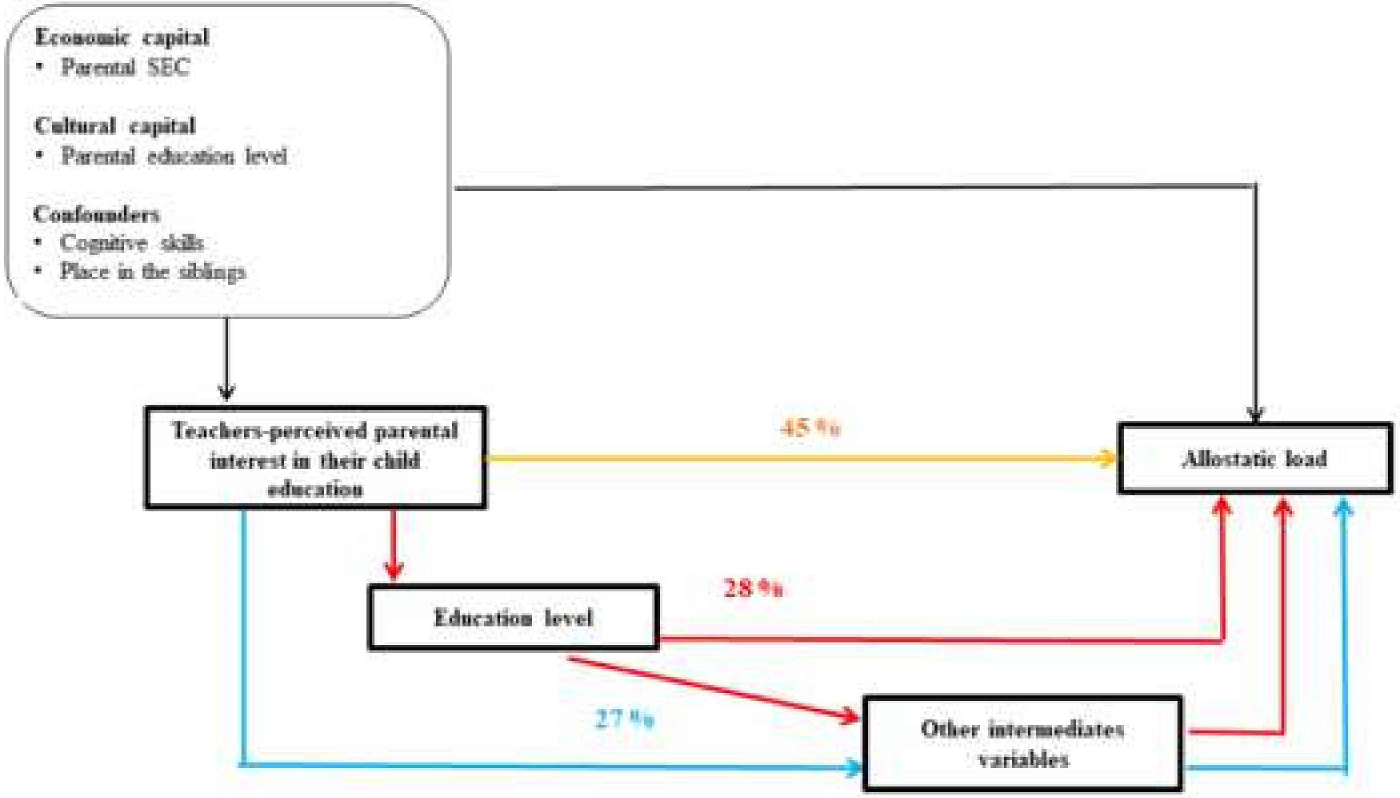

Click here to access/download
Supporting Information
S1_Table A.docx

Click here to access/download
Supporting Information
S2_File.docx

Click here to access/download
Supporting Information
S3_File.docx

Click here to access/download
Supporting Information
S4_Table B.docx
