# Supplementary material for: Could teacher-perceived parental interest be an important factor in understanding how education relates to later physiological health? A life course approach
Source: PLoS One. 2021 Jun 17;16(6):e0252518. doi: 10.1371/journal.pone.0252518 (PMC8211281; doi:10.1371/journal.pone.0252518)
Supplement: Attachment — Submitted filename: 1. Response to Reviewers.docx [file pone.0252518.s008.docx]

3rd May 2021

Dear editor and reviewer,

Thank you for the opportunity to submit a revised version of our manuscript titled « Could teacher-perceived parental interest be an important factor in understanding how education relates to later physiological health? A life course approach » to *PLOS One.* We appreciate the time and constructive feedback that you have provided on the manuscript to improve our paper.

Please find below our response to your comments and those made by reviewer 1. We have corrected two references on p.3 line 56: one was a duplicate and the other was less appropriate. We have also updated Tables 3 and 4 by harmonising the number of decimal results. To clarify English language issues the manuscript has been proofread by an anglophone.

With best wishes,

**Editor**

1. Personally, I usually recommend to abstain from all unnecessary abbreviations as the reader needs to remember these throughout the manuscript. So I would suggest to increase readability to say, "parents' interest in their child's education as perceived by teachers, mentioned as parental interest in the following", and speak of parental interest instead of PI; and of allostatic load instead of AL.

**Author response:** Thank you for this suggestion, we have amended the abbreviations throughout the text of the manuscript, to increase the readability, by using “parental interest” instead of PI, “allostatic load” instead of AL, and we have also added “Sense of personal control” instead if SOC.

2. I was pleased to see VanderWeele and Vansteelandt's method applied to test the putative mediators. If not absolutely necessary, I would remove references to Baron and Kenny and instead define mediators etc. in line with VanderWeele and Vansteelandt.

**Author response:** We have amended the manuscript to remove the reference to Baron and Kenny, and altered the paragraph on mediators in line with VanderWeele and Vansteelandt’s recommendation on page 11 line 240: “We defined the sets of mediators according to the temporal and causal assumptions of the life course framework. As such, we made a pragmatic methodological assumption about the temporal ordering of variables in order to examine the mediating pathways whereby the exposure variable (parental interest at age 7, 11 and 16) preceded the respondent’s education, which preceded the other intermediate variables (psychological distress, socioeconomic position and health behaviors)”.

3. I suggest to add to the abstract that the direct link between parental interest and allostatic load was completely mediated in men, but only partially mediated in women.

**Author response:** Thank you for this we have amended the abstract page 2 line 40.

4. The main set of results for men and women need to be carefully checked. For example, I would use p<0.001 or a manually adjusted p level to indicate significance, as otherwise family wise error rate (alpha inflation) would be a problem. Equally importantly, on p. 36, line 339 ff., it seems by adjusting for confounders, the main association between parental interest and allostatic load is rendered insignificant in men (the confidence interval includes the null, the p's are above 0.05). If I am reading this correctly, the results need to be reformulated to reflect the fact that the main association was not significant anymore in the adjusted models.

**Author response:** Thank you for pointing this out, we agree that the section highlighted was ambiguous. We have modified the formulation throughout the results section for the multivariate results for men to better reflect the non-significant association. Page 24 lines 345-347, after adjustment for prior confounder, the association between parental interest and allostatic load remained significant (Model 2, β= 0.18 [0.03; 0.32]; p-value= 0.015). However, from the model 3, the association was no longer significant (Model 3, β= 0.06 [-0.09; 0.21]; p-value=0.44). Therefore, we have added line 349 “which was no longer significant” and line 353 “After sequentially controlling for all time-ordered life course, SEP and health behaviors, the association between parental interest and allostatic load was attenuated and not statistically significant (Model 6, β= -0.01 [-0.16; 0.14]).”

5. Please also use . instead of , for the p values, confidence intervals (decimal points)

**Author response:** Thank you for alerting us to this error. We have replaced commas with decimal points in the main text and supplementary files.

6. p. 6 line 130 - there is an incomplete sentence (a few more missing periods throughout the manuscript, please check if ends of sentences are complete).

**Author response:** Thank you for pointing this omission out. We have carefully proofread the text for other omissions and for fluidity of language. Page 6 line 131: “PI is associated with allostatic load, and (ii) explore four pathways through which PI may be differentially embodied during childhood, adolescence and early adulthood, leading to physiological wear-and-tear, as measured by **allostatic load**” and page 13 line 285 “**See** S3 File for more information on the different steps of the analysis of estimated direct and indirect effects.”

**Reviewer**

1. This has improved compared to the first submission; however, I still think the authors need to improve on the sequential flow of the concept. I will also recommend that this paper is submitted to International Journal of Research and Scientific Innovation in Social sciences (RSIS) or a mental health journal as this is not a public health paper. It is kind of related to early childhood development or education and its answers some correlated questions to its impact on earning potential, midlife health and behavioural status but I still find it hard to conceptualize and put the jigsaw concisely.
   Line 49-50 please remove the “harsh material condition”
   Line 51-52 this sentence is unclear, …inequalities in total mortality of Who?
   The study design/methodology is not well-presented to explain the results. Interpretation of the results and the discussion are too wordy and are difficult to understand. It needs more careful proofreading.

**Author response:** Thank you for your helpful comments. We think that understanding how early life conditions in both the family and school environment may relate to later health are considerations of interest to a broad scientific and health audience, therefore we hope to pursue publication in Plos One.

Line 51-52 We replaced “harsh material condition” by disadvantaged material condition

Line 54-55 We added the additional point that it was total mortality of members of European cohorts.

We also have clarified some part of the methods section. Page 9 line 194 “To examine the relationship between parental interest and allostatic load, prior confounding variables potentially associated with both parental interest and allostatic load were added to the multivariable models. We selected variables most likely to be social or biological confounding factors from questionnaires completed during childhood by parents of cohort members, based on the literature.” Then the types of confounding factors were added such as “Socioeconomic confounders” line 199 or “Confounding factors related to family education” line 204.

Parts of the discussion section have been reorganized to make it easier to understand.

We look forward to hearing from you in due course regarding our submission and to respond to any further questions and comments you may have.

Sincerely,
